# Innate immune signaling in *Drosophila* shifts anabolic lipid metabolism from triglyceride storage to phospholipid synthesis to support immune function

**DOI:** 10.1371/journal.pgen.1009192

**Published:** 2020-11-23

**Authors:** Brittany A. Martínez, Rosalie G. Hoyle, Scott Yeudall, Mitchell E. Granade, Thurl E. Harris, J. David Castle, Norbert Leitinger, Michelle L. Bland

**Affiliations:** 1 Biomedical Sciences Graduate Program, University of Virginia, Charlottesville, VA, United States of America; 2 Department of Pharmacology, University of Virginia, Charlottesville, VA, United States of America; 3 Medical Scientist Training Program, University of Virginia, Charlottesville, VA, United States of America; 4 Department of Cell Biology, University of Virginia, Charlottesville, VA, United States of America; Department of Biosciences & Institute of Biotechnology, FINLAND

## Abstract

During infection, cellular resources are allocated toward the metabolically-demanding processes of synthesizing and secreting effector proteins that neutralize and kill invading pathogens. In *Drosophila*, these effectors are antimicrobial peptides (AMPs) that are produced in the fat body, an organ that also serves as a major lipid storage depot. Here we asked how activation of Toll signaling in the larval fat body perturbs lipid homeostasis to understand how cells meet the metabolic demands of the immune response. We find that genetic or physiological activation of fat body Toll signaling leads to a tissue-autonomous reduction in triglyceride storage that is paralleled by decreased transcript levels of the DGAT homolog *midway*, which carries out the final step of triglyceride synthesis. In contrast, Kennedy pathway enzymes that synthesize membrane phospholipids are induced. Mass spectrometry analysis revealed elevated levels of major phosphatidylcholine and phosphatidylethanolamine species in fat bodies with active Toll signaling. The ER stress mediator Xbp1 contributed to the Toll-dependent induction of Kennedy pathway enzymes, which was blunted by deleting AMP genes, thereby reducing secretory demand elicited by Toll activation. Consistent with ER stress induction, ER volume is expanded in fat body cells with active Toll signaling, as determined by transmission electron microscopy. A major functional consequence of reduced Kennedy pathway induction is an impaired immune response to bacterial infection. Our results establish that Toll signaling induces a shift in anabolic lipid metabolism to favor phospholipid synthesis and ER expansion that may serve the immediate demand for AMP synthesis and secretion but with the long-term consequence of insufficient nutrient storage.

## Author summary

Fighting infection requires that immune cells synthesize antimicrobial peptides and antibodies and carry out cellular processes like phagocytosis to destroy microbes and clear infected cells. During infection, metabolic processes support and direct immune function. Here, we use the fruit fly *Drosophila melanogaster* as a model system to understand the interaction between immunity and lipid metabolism. In *Drosophila* larvae, infection leads to tremendous production of antimicrobial peptides that destroy invading microbes. These peptides are made in the fat body, an organ that is also the site of fat storage. Activating the immune response reduces lipid storage but increases the production of phospholipids that form the membranes of organelles such as the endoplasmic reticulum. This organelle is the starting point for synthesis and secretion of antimicrobial peptides, and its volume is increased in response to immune activation. Shifting lipid metabolism to membrane phospholipid synthesis supports the immune response. However, this comes at the expense of the ability to withstand other types of stress such as desiccation at later stages in life. These findings are important because they suggest that some of the metabolic changes induced by fighting an infection may become detrimental if they are maintained over long periods of time.

## Introduction

Animals fight viral, microbial, and parasitic infections by activating humoral and cellular immune processes. Work in invertebrate and mammalian systems over many decades has identified the molecular pathways that lead from pathogen recognition to synthesis of effector proteins and activation of cellular processes that destroy microbes and infected cells. For example, presentation of pathogen-expressed molecules to conserved host pattern recognition receptors, such as *Drosophila* Toll and mammalian Toll-like receptors (TLRs), activates nuclear factor-κB (NF-κB) family transcription factors that direct expression of antimicrobial peptides, acute phase proteins, and cytokines. These effector proteins rupture microbial membranes, participate in opsonization, lysis and clotting reactions, and promote inflammation and activation of other immune cell types. In animals with adaptive immune systems, lymphocytes recognize pathogens via expression of recombinant receptors leading to immune cell clonal expansion, antibody secretion, and killing of infected cells.

Profound changes in host metabolism accompany the synthesis of antimicrobial peptides, acute phase proteins, cytokines, and antibodies as well as the induction of cellular processes such as phagocytosis and immune cell proliferation during the immune response. These metabolic changes support tolerance of and resistance to infection. At the whole-animal level, for example, rodents injected with lipopolysaccharide (LPS) or infected with *Escherichia coli* exhibit reductions in core temperature and oxygen consumption that drive disease tolerance [1,2]. At the level of individual cells, immune signaling shifts glucose metabolism from oxidative phosphorylation to aerobic glycolysis, a switch that promotes cytokine synthesis as well as survival, activation, and bactericidal functions of immune cell types in flies and mammals [3–6]. In dendritic cells stimulated with LPS, aerobic glycolysis drives fatty acid synthesis that underlies expansion of the endoplasmic reticulum (ER) [7]. Changes in lipid metabolism promote secretory function in multiple immune cell types. Upon simulation with LPS, mouse B cells differentiate into plasma cells that synthesize and secrete antibodies; this is accompanied by increased membrane phospholipid synthesis and ER expansion [8,9]. These changes are dependent on splicing of the *X-box binding protein 1* (*Xbp1*) mRNA, leading to a mature transcript that encodes the Xbp1 transcription factor, a key mediator of the unfolded protein response [10,11]. Similarly, in macrophages, phosphatidylcholine synthesis and Xbp1 activation are necessary for maximal levels of cytokine secretion in response to infection or LPS stimulation [12,13]. The mechanisms linking immune signaling with regulation of carbohydrate and lipid metabolic pathways, whether in the physiological response to infection or during pathological disease states characterized by chronic inflammation remain unclear in many cases.

Fighting infection is energetically demanding, and, as in mammals, activation of innate immune signaling in *Drosophila* alters metabolism. Flies respond to septic injury with Gram-positive bacteria or fungi by activating a conserved Toll-NF-κB pathway that stimulates synthesis and secretion of micromolar quantities of antimicrobial peptides (AMPs) into hemolymph [14,15]. AMPs such as Drosomycin fight infection by disrupting microbial membranes and inhibiting fungal spore germination [16,17]. The major source of circulating AMPs in infected *Drosophila* larvae is the fat body, an organ that coordinates not only the humoral immune response via activation of the Toll and Imd signaling pathways but also nutrient storage and animal growth [14,18]. Metabolism and growth are regulated by *Drosophila* insulin-like peptides that bind to insulin receptors on fat body cells, leading to activation of Akt and mTOR to stimulate protein synthesis, cell growth, and storage of dietary sugar as triglycerides and glycogen via highly-conserved metabolic pathways [19]. The coordination of immune, growth, and metabolic pathways in the same cells of the fat body and the sequence and functional conservation of these pathways between flies and mammals make *Drosophila* an attractive model for the study of immunometabolism at the scale of whole-animal physiology. Infection of adult flies with the intracellular bacterial pathogen *Mycobacterium marinarum* leads to a progressive depletion of whole-animal triglyceride stores along with lipid accumulation in phagocytes that harbor mycobacteria [20,21]. Colonization of adult fat body cells with the intracellular parasite *Tubulinosema ratisbonensis* also impairs triglyceride storage, directing host fatty acids to fuel parasite growth [22]. Lipid storage defects can be elicited by genetic activation of the Toll and Imd pathways, indicating that metabolic changes are dictated not only by pathogen interaction but also by signaling from the host immune system. Activation of the Imd pathway in larval fat body results in decreased triglyceride levels and impaired whole-animal growth [23]. Expression of a constitutively-active Toll receptor, Toll^10b^, in larval fat body inhibits whole-animal growth, disrupts insulin signaling in fat body, and reduces triglyceride storage [24–26].

While the signaling events that lead from pathogen recognition to immune effector production are well understood, the mechanisms that underlie altered lipid metabolism in response to immune signaling and the short- and long-term consequences of such metabolic changes remain unclear. To address this, we genetically activated Toll signaling in the larval fat body or infected larvae with the Gram-positive bacteria *Enterococcus faecalis* and assessed expression of enzymes that carry out *de novo* lipogenesis. We find that the decrease in triglycerides caused by active Toll signaling is mirrored by a selective decrease in expression of enzymes that carry out the two final steps of *de novo* triglyceride synthesis: the phosphatidic acid phosphatase Lipin and the diacylglycerol transferase homolog midway. Enzymes that carry out early steps of fatty acid synthesis are unchanged or elevated in response to Toll signaling, leading us to investigate other fates of fatty acids. We find that Toll signaling induces a coordinated increase in expression of enzymes in the Kennedy phospholipid synthesis pathway and elevated levels of the membrane phospholipids phosphatidylethanolamine and phosphatidylcholine. The transcription factor Xbp1 participates in the induction of Kennedy pathway enzymes by Toll receptor activation, suggesting a contribution of ER stress to this phenotype. Indeed, the ER is expanded and dilated in fat body cells with active Toll signaling. Deletion of genes encoding AMPs to reduce the secretory burden caused by Toll signaling blunts induction of Kennedy pathway enzymes, but not splicing of *Xbp1*, in immune-activated fat body cells. Blocking expression of two key Kennedy pathway enzymes, Pcyt1 and eas, impairs AMP expression and clearance of bacteria in response to infection. Our results suggest a mechanism by which phospholipid biosynthesis, induced downstream of Toll receptor activation, supports ER expansion to sustain AMP production during the immune response. Our data also indicate that a long-term consequence of this metabolic switch that directs fatty acids from neutral lipid storage toward phospholipid synthesis is reduced survival during stress conditions.

## Results

### Toll signaling in fat body acts in a tissue-autonomous manner to disrupt nutrient storage

**Fig 1:**
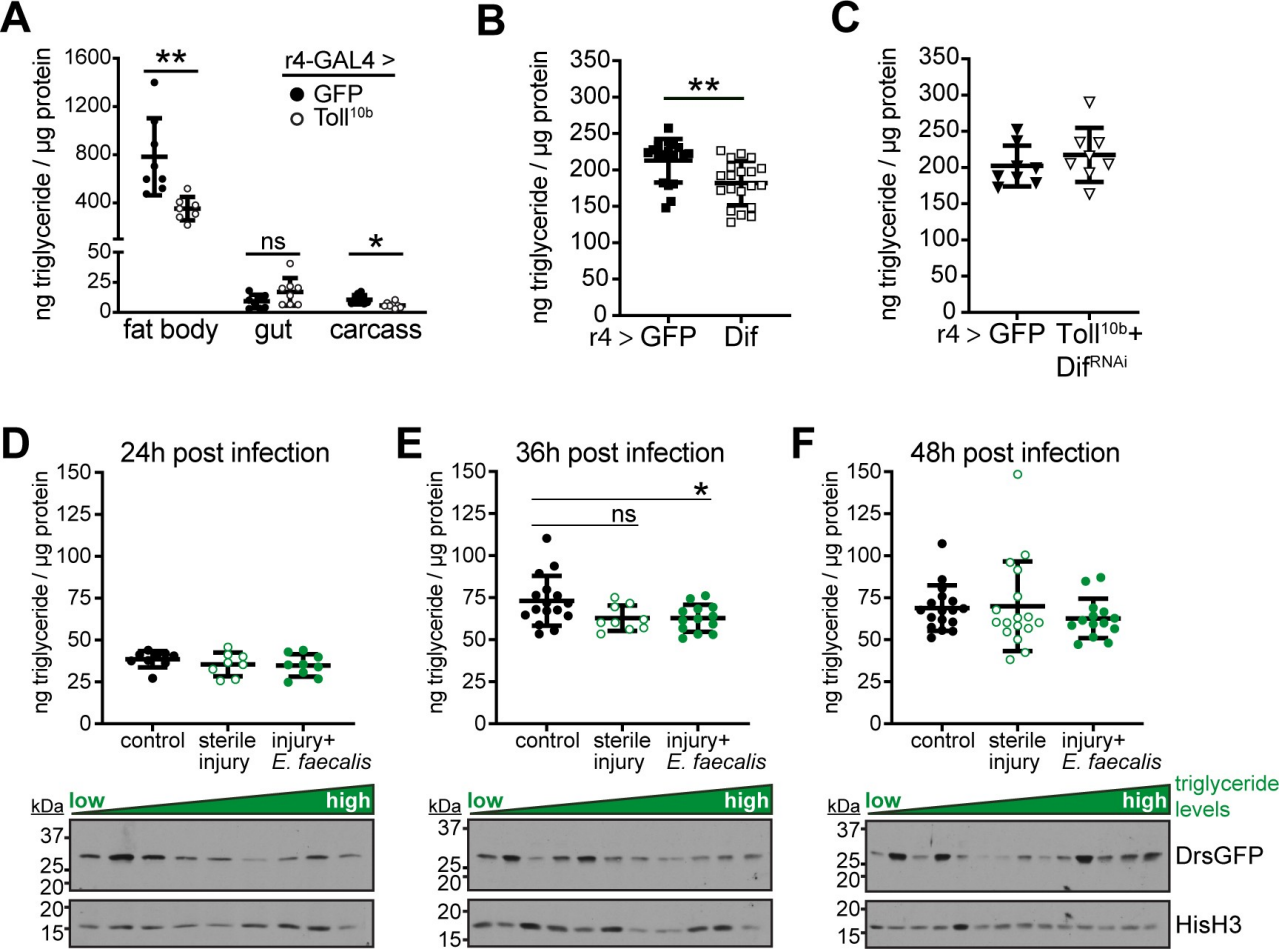
Toll signaling in the third instar larval fat body reduces triglyceride storage in a tissue-autonomous manner. For A-C, r4-GAL4 was used to drive indicated transgenes in fat body, and triglyceride levels were measured in whole larvae or dissected organs and normalized to protein levels. (**A**) Triglyceride levels in fat body, gut, or carcass, n = 7-8/group. *p = 0.0118 and **p = 0.0047 versus GFP. (**B**) Whole-animal triglyceride levels in larvae expressing GFP or Dif in fat body, n = 18-20/group. **p = 0.0034 versus GFP. (**C**) Whole-animal triglyceride levels in larvae expressing GFP or Toll^10b^+Dif^RNAi^ in fat body, n = 8/group. (**D-F**) Top: whole-animal triglyceride levels, normalized to protein, in controls and larvae subjected to sterile injury or injury with *E*. *faecalis* infection. Bottom: Western blot analysis of DrsGFP reporter levels in triglyceride lysates from larvae infected with *E*. *faecalis*. Histone H3 (HisH3) is shown as a loading control. (**D**) 24h post infection, n = 8-9/group. (**E**) 36h post infection, n = 9-16/group. *p = 0.0393. (**F**) 48h post infection, n = 14-17/group. Data are presented as means ± SD. p values were determined by Student’s unpaired t test (A-C) and one-way ANOVA with Dunnett’s multiple comparison test (D-F).

Genetic activation of Toll signaling in the larval fat body, via r4-GAL4-dependent expression of a constitutively-active Toll^10b^ transgene, reduces whole-animal triglyceride levels, although the mechanism for reduced lipid storage has been unclear [24,25]. The fat body stores the bulk of triglycerides in fruit fly larvae, and we find that activating Toll signaling in larval fat body, via r4-GAL4 driven expression of Toll^10b^, decreased late third instar fat body triglyceride levels by 55%. Expression of Toll^10b^ in fat body led to negligible changes in gut and carcass triglycerides compared with tissues from control larvae expressing GFP in fat body (Fig 1A). We also note, as expected, that the fat body stored 70–85 times more triglyceride than the gut or the carcass, which comprises cuticle, muscle, oenocytes, imaginal discs, and trachea. Throughout the larval third instar, animals with active fat body Toll signaling consistently stored less triglyceride than GFP-expressing controls (S1 Fig). Reduced triglyceride storage in animals with active Toll signaling persisted to the white prepupal stage, a well-defined developmental endpoint that follows the cessation of feeding, indicating that low triglyceride levels are not due to a developmental delay. To understand whether the decrease in triglycerides elicited by innate immune signaling is transcriptionally regulated, we manipulated the NF-κB homolog Dif. Dif is activated by Toll signaling and binds directly to promoters of genes encoding antimicrobial peptides (AMPs) such as *Drosomycin*, leading to high levels of AMP production in fat body in response to infection with fungi or Gram-positive bacteria such as *Enterococcus faecalis* (S2 Fig). Elevated expression of Dif in larval fat body phenocopied Toll^10b^, leading to a decrease in whole-animal triglyceride levels (Fig 1B). However, loss of Dif in fat bodies with active Toll signaling rescued triglyceride storage (Fig 1C).

We used a second fat body driver, cg-GAL4, to validate our results with r4-GAL4. The r4-GAL4 and cg-GAL4 drivers induce UAS transgene expression in larval fat body, and each exhibits restricted expression in other organs (S3A Fig). Expression of Toll^10b^ under control of cg-GAL4 induces fat body expression of the antimicrobial peptide gene *Drosomycin* (S3B Fig) and lowers whole-animal triglyceride levels by 41% compared with GFP controls (S3C Fig).

To determine whether infection disrupts lipid storage in a manner similar to genetic activation of Toll signaling, we challenged early third instar larvae with the Gram-positive, pathogenic bacteria *Enterococcus faecalis* [27] and monitored triglyceride levels beginning at 24 hours after infection. We chose this time course because larval triglyceride storage increases three- to four-fold from the beginning of the third instar at 72h after egg lay (AEL) to the end of this stage 48 hours later (refer back to S1 Fig). Because injury also activates the innate immune response in a manner dependent on Toll pathway components including MyD88 [28,29], we included mock-infected controls that were punctured without bacterial exposure. At 24 hours post infection, triglyceride levels were equivalent among control larvae, larvae with sterile injury, and larvae with injury and bacterial infection (Fig 1D). At 36 hours post infection, control animals had doubled the level of stored triglyceride relative to the 24 hour time point (equivalent to 96h AEL). However, triglyceride accumulation was significantly reduced by 14% in infected larvae. Triglyceride levels were similarly decreased in larvae subjected to sterile injury, but this difference failed to reach statistical significance (p = 0.0701) (Fig 1E). By 48 hours post infection, triglyceride levels were again equivalent among groups, indicating a transient block of triglyceride accumulation in response to infection (Fig 1F). Larvae in this experiment carried DrsGFP, a reporter of Toll signaling consisting of the *Drosomycin* promoter fused to the GFP coding sequence [30]. Western analysis of the whole-animal lysates used for triglyceride measurements showed that at both 24 and 36 hours post infection, animals with the lowest triglyceride levels had the highest levels of DrsGFP expression (bottom panels, Fig 1D and 1E). By 48 hours post infection, when triglyceride levels were equivalent with control animals, there was no clear relationship between lipid storage and DrsGFP expression (bottom panel, Fig 1F).

A major role of stored triglycerides is to provide energy during starvation. We asked whether decreased triglyceride storage caused by larval fat body Toll signaling affected starvation resistance in adult flies that expressed GFP or Toll^10b^ in fat body throughout the larval and pupal stages under control of r4-GAL4. We saw no difference in starvation sensitivity in female or male flies of either genotype that were given water (S4A Fig). However, when we subjected flies to the combined stressor of starvation and desiccation, we observed a significant decrease in stress resistance in flies that expressed Toll^10b^ in fat body compared with GFP-expressing controls (S4B Fig). Median survival time during starvation without water was decreased by 33% and 43%, respectively, in male and female flies expressing Toll^10b^ compared with GFP-expressing controls. Decreased resistance to desiccation is consistent with a failure to waterproof the cuticle, and recent work shows that about half of the triglyceride stored by the larval fat body is used for this purpose [31].

**Fig 2:**
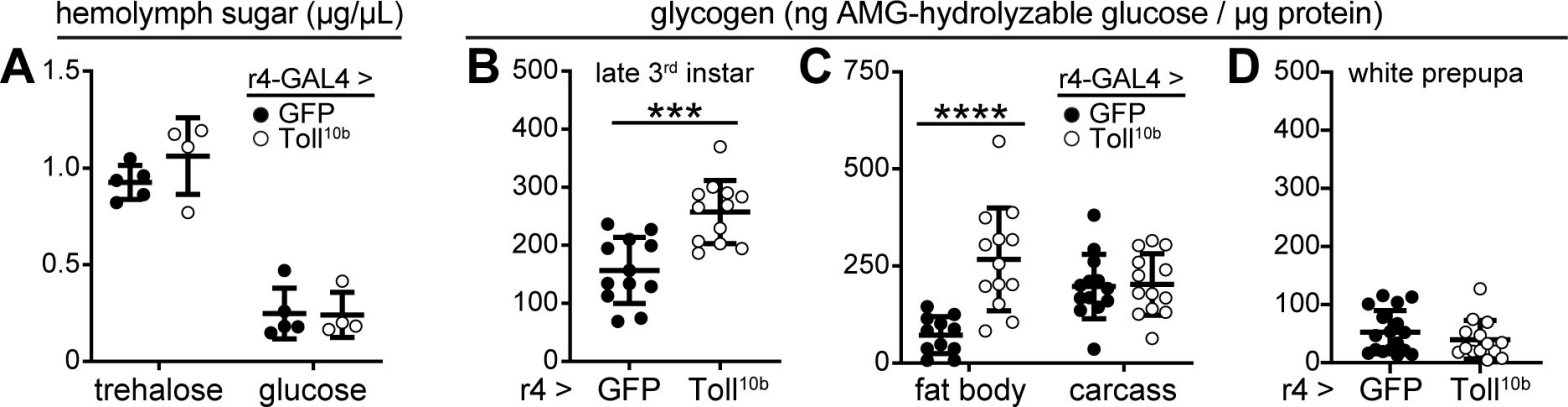
Toll signaling leads to increased fat body glycogen storage in the larval stage. (**A**) Hemolymph glucose and trehalose levels in mid-third instar larvae expressing GFP or Toll^10b^ in fat body under control of r4-GAL4, n = 4-5/group. (**B**) Late third instar whole-animal glycogen levels, normalized to protein, n = 12/group. ***p = 0.0002 versus GFP. (**C**) Glycogen levels in fat body and body wall, normalized to protein, from late third instar larvae expressing GFP or Toll^10b^ in fat body, n = 11-13/group, ****p < 0.0001 versus GFP. (**D**) White prepupal whole-animal glycogen levels, normalized to protein, n = 14-17/group. Data are presented as means ± SD. p values were determined by Student’s unpaired t test.

We investigated the mechanism for reduced triglyceride storage in the immune-activated fat body by examining levels of circulating glucose, the substrate for *de novo* fatty acid and triglyceride synthesis. Trehalose, a disaccharide composed of two glucose molecules, is the major circulating sugar in fruit flies. Hemolymph trehalose and glucose levels were equivalent in larvae expressing GFP or Toll^10b^ in fat body (Fig 2A), suggesting that altered substrate availability does not account for reduced triglyceride storage. Another fate of glucose is storage as the branched polysaccharide glycogen. Surprisingly, whole-animal glycogen levels were increased in third instar larvae with active Toll signaling in fat body (Fig 2B). To better understand this phenotype, we measured glycogen in two tissues that are the major sites of glycogen storage in fly larvae, fat body and body wall muscles [32]. We observed a 3.7-fold increase in glycogen levels in fat body but no change in glycogen in the carcass, containing the body wall musculature, in animals with active fat body Toll signaling compared with controls (Fig 2C). Glycogen levels in white prepupae were reduced from third instar levels and were equivalent in animals expressing GFP or Toll^10b^ in fat body (Fig 2D). Together these data show that triglyceride and glycogen storage are regulated differently by innate immune signaling and that an impairment in lipid storage correlates with reduced survival in response to desiccation stress in the adult stage. Because genetic or physiological activation of the Toll pathway lowers triglyceride storage, our next step was to determine the molecular mechanisms underlying this phenotype.

### Toll signaling negatively regulates dedicated steps of triglyceride synthesis

**Fig 3:**
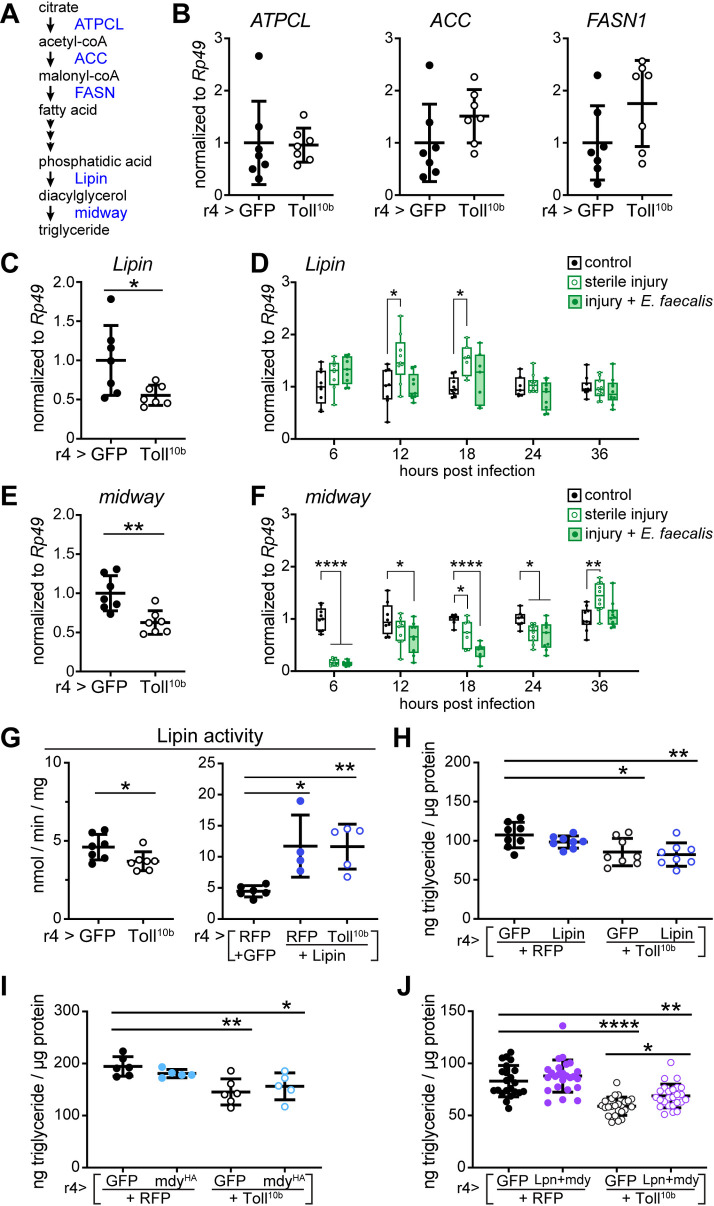
Reduced expression of *Lipin* and the DGAT homolog *midway* correlate with low triglyceride levels in larvae with active Toll signaling. (**A**) Schematic representation of the *de novo* lipogenic pathway leading to triglyceride synthesis. (**B-F**) Total RNA was extracted from late third instar larval fat bodies expressing the indicated transgenes under control of r4-GAL4 or fat bodies from control, injured, and infected larvae dissected at indicated times following sterile injury or infection with *Enterococcus faecalis* early in the third instar stage. All transcript levels were measured by RT-qPCR and normalized to *Rp49*. (**B**) *ATPCL*, *ACC*, and *FASN1* transcripts in late third instar larval fat bodies expressing GFP or Toll^10b^, n = 7/group. (**C**) *Lipin* mRNA levels in late third instar larval fat bodies expressing GFP or Toll^10b^, n = 7/group. *p = 0.0259 versus GFP. (**D**) *Lipin* mRNA levels in third instar larval fat bodies at 6–36 hours post infection, n = 7-10/group. *p ≤ 0.0133 versus uninfected controls. (**E**) *midway* mRNA levels in late third instar larval fat bodies expressing GFP or Toll^10b^, n = 7/group. **p = 0.0031 versus GFP. (**F**) *midway* mRNA levels in third instar larval fat bodies at 6–36 hours post infection, n = 7-10/group. *p ≤ 0.0350, **p ≤ 0.0017, and ****p < 0.0001 versus uninfected controls. (**G**) Lipin activity in fat bodies from late third instar larvae expressing GFP or Toll^10b^ (left) and RFP+GFP, RFP+Lipin, or Toll^10b^+Lipin (right) under control of r4-GAL4, n = 4-7/group. *p ≤ 0.0179 versus GFP (left) or RFP+GFP (right) and **p = 0.0097 versus RFP+GFP. (**H**) Triglyceride levels in late third instar larvae expressing Toll^10b^ with or without a wild type Lipin transgene in fat body, n = 8/group. *p = 0.0164 and **p = 0.0053 versus RFP+GFP. (**I**) Triglyceride levels in late third instar larvae expressing Toll^10b^ with or without a wild type mdy^HA^ transgene in fat body, n = 5-6/group. *p = 0.0193 and **p = 0.0020 versus RFP+GFP. (**J**) Triglyceride levels in late third instar larvae expressing Toll^10b^ with or without wild type Lipin and mdy^HA^ transgenes in fat body, n = 22-25/group. **p = 0.0016 and ****p < 0.0001 versus RFP+GFP, and *p = 0.0193 versus GFP+Toll^10b^. Data are shown as means ± SD. p values were determined by Student’s t test (B, C, E and G, left panel) and one-way ANOVA with Dunnett’s (D, F, H, I) or the Tukey-Kramer (G, right panel, J) multiple comparisons test.

*De novo* lipogenesis is controlled in part by transcriptional regulation of genes encoding lipogenic enzymes [33]. The enzymes ATP citrate lyase (ATPCL), Acetyl-CoA carboxylase (ACC), and Fatty acid synthase 1 (FASN1) synthesize fatty acids from glucose-derived citrate (Fig 3A). Fat body-specific expression of *ATPCL* was unchanged by activation of Toll signaling. Transcripts encoding *ACC* and *FASN1* were elevated by 51–75% in Toll^10b^-expressing fat bodies compared with controls, but these differences were not statistically significant (Fig 3B). In contrast, Toll signaling in fat body, induced by transgenic expression of Toll^10b^ with r4-GAL4 or cg-GAL4 led to a 39–45% reduction in transcripts encoding *Lipin*, a phosphatidic acid phosphatase that synthesizes diacylglycerol from phosphatidic acid (Figs 3C and S3D). However, unlike the results with chronic, high-level signaling induced by Toll^10b^ transgene expression, physiological activation of Toll signaling did not reduce *Lipin* mRNA levels after sterile injury or infection (Fig 3D).

Genetic activation of fat body Toll signaling reduced expression of the diacylglycerol acyltransferase (DGAT) homolog *midway*, an enzyme that carries out the final step in triglyceride synthesis, by 38% compared with controls (Fig 3E). In contrast, we saw no change in *midway* expression when Toll^10b^ was expressed under cg-GAL4 control (S3D Fig). In response to sterile injury or infection with *Enterococcus faecalis*, however, fat body levels of *midway* were sharply reduced early and this decrease persisted for 24 hours following infection (Fig 3F).

Lipin and midway are necessary for triglyceride storage. Knockdown of either enzyme in fat body or loss of midway in the whole animal decreased triglyceride levels (S5A–S5C Fig), consistent with previous findings [34–37]. Elevated fat body expression of Lipin or midway was not sufficient to increase triglyceride accumulation in otherwise wild type larvae (S5D–S5F Fig). However, fat body-specific expression of a UAS-midway transgene restored triglycerides in flies heterozygous for the *mdy*^*QX25*^ null mutation (S5G Fig).

We performed transgenic rescue experiments to determine whether forced expression of Lipin or midway would restore triglyceride accumulation in animals with active fat body Toll signaling. Consistent with decreased *Lipin* transcript levels, we find a 20% decrease in Lipin activity in extracts of fat bodies expressing Toll^10b^ compared with controls expressing GFP (Fig 3G, left). Forced expression of a wild type Lipin transgene in fat body increased Lipin activity 2.6-fold in extracts from fat bodies co-expressing RFP or Toll^10b^ compared with control extracts (Fig 3G, right). However, elevated Lipin expression and enzyme activity failed to rescue low triglyceride levels in larvae co-expressing Toll^10b^ (Fig 3H). The ability of Lipin overexpression to rescue Lipin activity but not triglyceride storage raised the possibility that midway was limiting for triglyceride storage during chronic Toll activation in the fat body. However, similar to results found with Lipin, co-expression of a wild type midway transgene with Toll^10b^ did not rescue triglyceride levels (Fig 3I).

We considered the possibility that when either Lipin or midway is overexpressed, the other enzyme becomes rate limiting in *de novo* lipogenesis. To test this, we generated flies co-expressing both Lipin and midway under control of the r4-GAL4 fat body driver, leading to 10-50-fold higher transcript levels of each enzyme even in the presence of Toll^10b^ (S5H Fig). Co-expression of Lipin and midway in fat body does not increase whole-animal triglyceride levels compared with controls expressing RFP and GFP (Fig 3J). In contrast, co-expression of Lipin and midway with Toll^10b^ in fat body led to a significant, 16% increase in triglyceride levels compared with animals expressing Toll^10b^ alone (Fig 3J). The failure of Lipin and midway co-expression to fully rescue triglyceride levels is consistent with the possibility that fatty acids are being diverted to another purpose in cells with active Toll signaling and that elevated flux through this second pathway could prevent triglyceride accumulation.

### Innate immune signaling induces phospholipid synthesis

**Fig 4:**
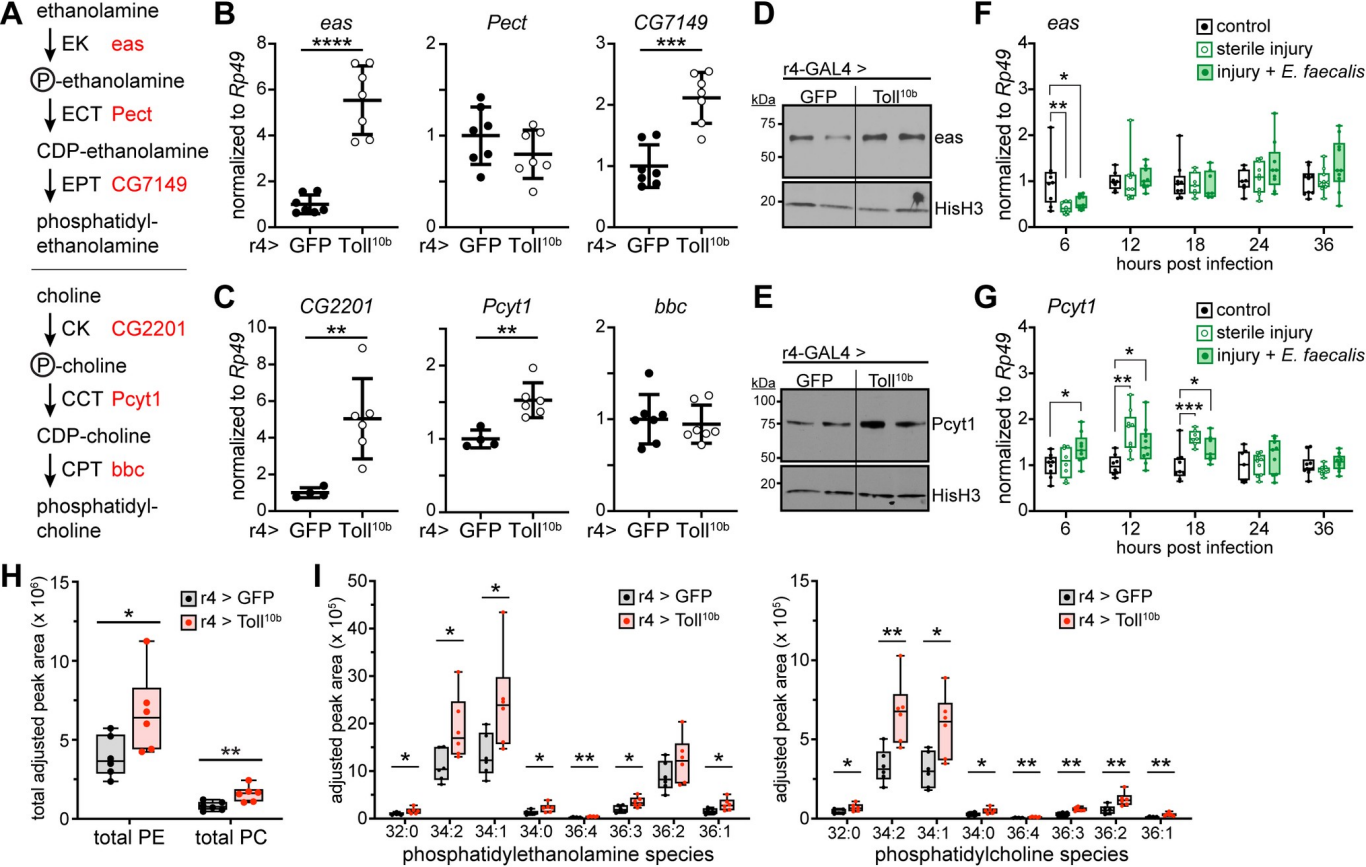
Toll signaling in fat body leads to increased levels of phospholipid synthesis enzymes and elevated membrane phospholipids. (**A**) Schematic representation of phosphatidylethanolamine (PE) and phosphatidylcholine (PC) synthesis via the Kennedy pathway. (**B**) Late third instar fat body levels of *eas*, *Pect*, and *CG7149* transcripts, normalized to *Rp49*, n = 7/group. ***p = 0.0002 and ****p = 0.0001 versus GFP. (**C**) Late third instar fat body levels of *CG2201*, *Pcyt1*, and *bbc* transcripts, normalized to *Rp49*. n = 7/group. **p ≤ 0.0071 versus GFP. (**D,E**) Western blot analysis of eas (D) and Pcyt1 (E) protein levels in fat bodies expressing GFP or Toll^10b^ under control of r4-GAL4. Histone H3 levels are shown as loading controls. (**F,G**) *eas* (F) and *Pcyt1* (G) mRNA levels, normalized to *Rp49*, in third instar larval fat bodies from uninfected controls, larvae subjected to sterile injury, and larvae infected with *Enterococcus faecalis* at 6–36 hours post infection, n = 7-10/group. *p ≤ 0.0496, **p ≤ 0.0035, and ***p = 0.0009 versus uninfected controls. (**H**) Mass spectrometry analysis of total phosphatidylethanolamine levels (PE, left) and phosphatidylcholine levels (PC, right) in lipid extracts from late third instar larval fat bodies expressing GFP or Toll^10b^, n = 6/group. *p = 0.0417 and **p = 0.0080 versus GFP. (**I**) Mass spectrometry analysis of PE (left) and PC species (right) in lipid extracts from late third instar larval fat bodies expressing GFP or Toll^10b^, n = 6/group. *p < 0.05, **p < 0.01 versus GFP. Data are shown as means ± SD. p values were determined by Student’s unpaired t test (B, C, H, I) and one-way ANOVA with Dunnett’s multiple comparisons test (F, G).

Given that *ATPCL*, *ACC*, and *FASN1* are expressed at normal to elevated levels in fat bodies with active Toll signaling, we considered that fatty acids produced by the actions of these enzymes might be used for purposes other than triglyceride storage during the immune response. A major fate of fatty acids in cells is phospholipid synthesis via the Kennedy pathway (Fig 4A). We assessed transcript levels of Kennedy pathway homologs in fat bodies expressing GFP or Toll^10b^ under control of r4-GAL4 or cg-GAL4. We find elevated transcript levels of both the ethanolamine kinase *easily shocked* (*eas*) and the diacylglycerol ethanolaminephosphotransferase *CG7149* in fat bodies with active Toll signaling compared with controls (Figs 4B and S3E). *Phosphocholine cytidylyltransferase 1* (*Pcyt1*) was also induced by Toll signaling (Figs 4C and S3E), while the choline kinase homolog *CG2201* was inconsistently increased by fat body Toll pathway activity (compare data from independent experiments shown in Figs 4C and S6A). Protein levels of eas and Pcyt1 were noticeably induced in fat bodies with active Toll signaling (Figs 4D, 4E and S3F). In response to sterile injury or infection with *Enterococcus faecalis*, fat body levels of *eas* were largely unchanged, although we note decreased levels in both groups at 6 hours post infection (Fig 4F). However, *Pcyt1* transcript levels were induced in fat bodies during the first 18 hours following sterile injury or infection and then decreased to uninfected levels (Fig 4G). Other enzymes in the Kennedy pathway, such as *Phosphoethanolamine cytidylyltransferase* (*Pect*), the diacylglycerol cholinephosphotransferase *bb in a boxcar* (*bbc*) (Fig 4B and 4C), *Phosphocholine cytidylyltransferase 2* (*Pcyt2*) and the diacylglycerol ethanolaminephosphotransferase *CG33116* (S6B Fig) were not induced by Toll signaling. We note that our previously published RNA-Seq data [26] show much higher expression of *Pcyt1* compared with *Pcyt2* in larval fat body (S6C Fig).

To determine the functional consequences of increased *eas*, *CG7149*, *CG2201*, and *Pcyt1* expression in fat bodies with active Toll signaling, we assessed phospholipid levels in fat body. Using thin layer chromatography (TLC), we found increases in total phosphatidylethanolamine (PE) and phosphatidylcholine (PC) in fat bodies expressing Toll^10b^ compared with controls expressing GFP (S6D and S6E Fig). As a more sensitive assay, we used mass spectrometry to measure levels of the major PE and PC species in fat body [38–40]. In agreement with our TLC results, we found increased total levels of PE and PC in fat bodies expressing Toll^10b^ compared with controls (Fig 4H). We note that our TLC and mass spectrometry data show higher levels of PE than PC in both genotypes, as is true of membrane phospholipid composition in flies, and opposite to the pattern in mammals, where PC predominates [41]. Levels of most major PE and PC species were increased 1.5- to 2-fold in Toll^10b^-expressing fat bodies compared with controls (Fig 4I). These data suggest that increased expression of Kennedy pathway enzymes translates to substantial changes in phospholipid metabolism in response to immune signaling.

### Transcriptional control of Kennedy pathway enzymes in cells with active Toll signaling

We asked whether the canonical downstream transcription factor in the Toll signaling pathway, the NF-κB family member Dif, was sufficient to regulate expression of Kennedy pathway enzymes. Elevated expression of Dif in fat body using the r4-GAL4 driver induced expression of both *eas* and *Pcyt1* (S7A Fig). Dif also phenocopied Toll^10b^ in the regulation of the lipogenic genes *Lipin* and *midway*, leading to reductions in transcript levels for both of these genes (S7B Fig). These lipid metabolic enzymes are not known to be direct transcriptional targets of Dif, so we asked whether other transcription factors might act downstream of Toll and Dif to regulate lipid metabolism.

We investigated the role of two transcription factors known to participate in phospholipid homeostasis–Sterol regulatory element binding protein (SREBP) and X-box binding protein-1 (Xbp1)–in the elevated expression of Kennedy pathway enzymes in fat bodies with active Toll signaling. In flies, as in mammals, the transcription factor SREBP is a key regulator of *de novo* lipogenesis [42]. We find that fat bodies expressing Toll^10b^ exhibit elevated expression of SREBP mRNA and protein (S8A and S8B Fig). However, fat body-specific knockdown of SREBP did not affect either basal or Toll signaling-induced expression of *eas*, *CG7149*, or *CG2201*. Although basal expression of *Pcyt1* was reduced nearly three-fold by loss of SREBP, activation of Toll signaling led to 1.8-fold induction of *Pcyt1* in fat bodies regardless of SREBP levels (S8C Fig).

**Fig 5:**
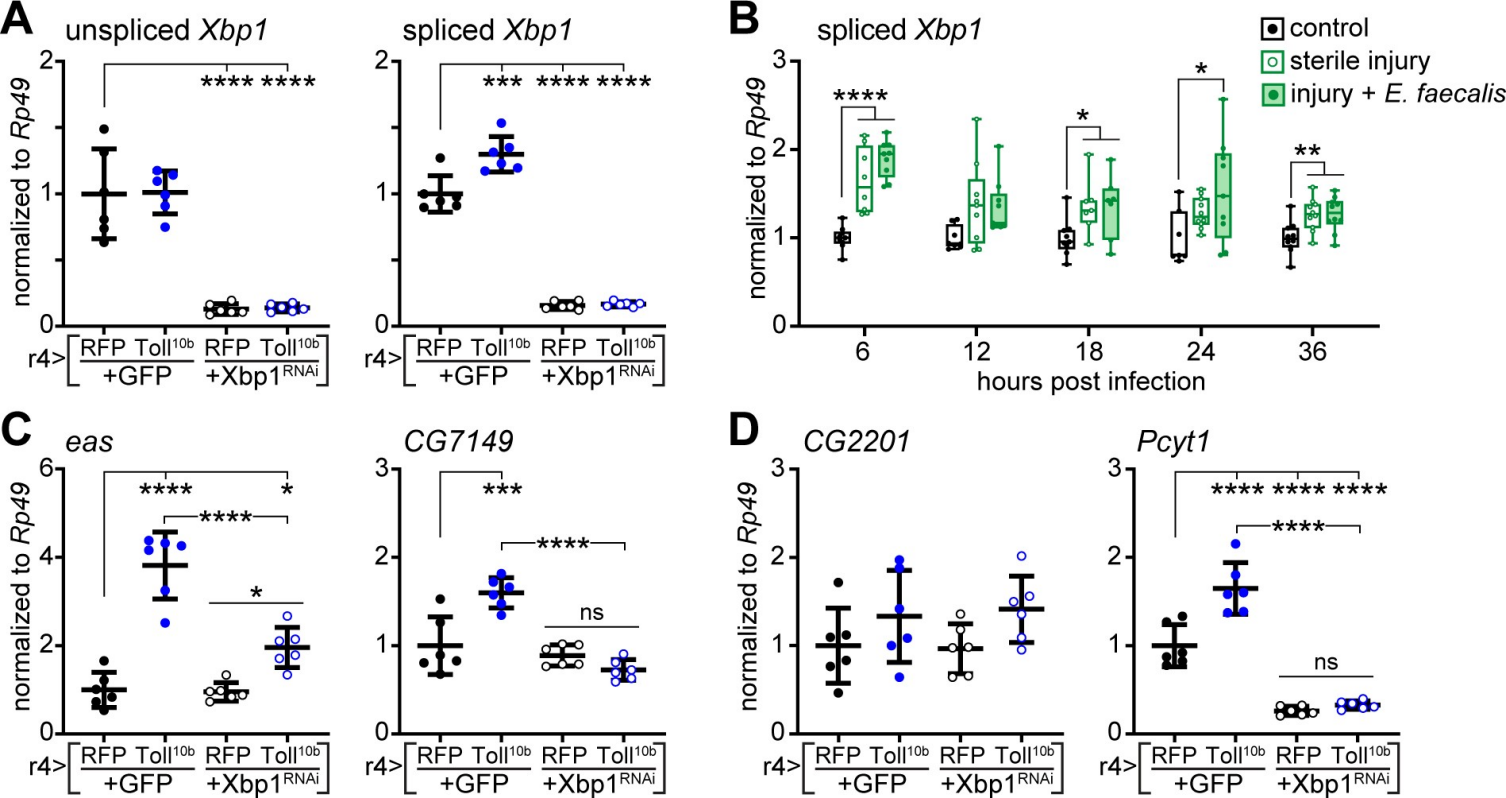
Toll signaling induces Kennedy pathway enzymes in a Xbp1-dependent manner. (**A**) Transcript levels of unspliced (left) and spliced (right) *Xbp1* were measured by RT-qPCR in late third instar larval fat bodies with GAL80ts-mediated induction of Toll^10b^ with or without Xbp1^RNAi^ for 24 hours at 30°C, n = 6/group. ***p = 0.0001 and ****p < 0.0001 versus fat bodies acutely expressing RFP+GFP. (**B**) Transcript levels of spliced *Xbp1*, normalized to *Rp49*, in third instar larval fat bodies from uninfected controls, larvae subjected to sterile injury, and larvae infected with *Enterococcus faecalis* at 6–36 hours post infection n = 7-10/group. *p ≤ 0.0484, **p ≤ 0.0081, and ****p < 0.0001 versus uninfected controls. (**C, D**) Transcript levels of indicated genes in late third instar larval fat bodies with GAL80ts-mediated induction of Toll^10b^ with or without Xbp1^RNAi^ for 24 hours at 30°C, n = 6/group. **p = 0.0087, ***p = 0.0001, and ****p < 0.0001 versus fat bodies acutely expressing RFP+GFP. Data are presented as means ± SD. p values were determined by one-way ANOVA with Dunnett’s (A, B) or the Tukey-Kramer multiple comparisons test (C, D).

The unfolded protein response mediator Xbp1 regulates *de novo* lipogenesis of membrane phospholipids to permit ER expansion [43]. Xbp1 is activated by Ire1-mediated splicing [44,45], and fat bodies expressing Toll^10b^ under control of r4-GAL4 (Fig 5A) or cg-GAL4 (S3G Fig) exhibit elevated levels of spliced *Xbp1* compared with controls. Levels of unspliced *Xbp1* were largely unchanged by expression of Toll^10b^ in fat body (Fig 5A). Forced expression of Dif in fat body also induced Xbp1 splicing, and loss of Dif in fat bodies expressing Toll^10b^ prevented the increase in spliced *Xbp1* (S7C Fig). In larvae subjected to sterile injury or infection with *Enterococcus faecalis*, levels of spliced *Xbp1* were increased between 1.3- to 1.9-fold compared with uninfected controls at 6, 18, 24, and 36 hours following infection (Fig 5B). To test the role of Xbp1 in regulation of Kennedy pathway enzymes by innate immune signaling, we attempted Xbp1 loss-of-function experiments in fat bodies co-expressing Toll^10b^. *Xbp1* null mutants die as second instar larvae [46], and chronic knockdown of *Xbp1* in larval fat body via r4-GAL4 strongly inhibits whole-animal growth. However, acute induction of both Toll^10b^ and Xbp1^RNAi^ transgenes in fat body using temperature-sensitive TubGAL80ts to restrict r4-GAL4 activity to a 24-hour period led to a near-complete loss of *Xbp1* transcripts (Fig 5A). Knocking down *Xbp1* blunted Toll^10b^-dependent induction of *eas* and blocked Toll^10b^-dependent induction of *CG7149* (Fig 5C). Acute loss of *Xbp1* in fat body reduced basal levels of *Pcyt1* transcripts. Acute expression of Toll^10b^ induced *Pcyt1* 1.6-fold compared with controls, but only 1.2-fold in fat bodies with simultaneous loss of *Xbp1* (Fig 5D). We did not observe induction of *CG2201* in this experiment, perhaps due to the acute nature of Toll^10b^ expression (Fig 5D). Finally, acute loss of *Xbp1* blunted induction of *SREBP* and the AMP *Drs* by Toll signaling (S8E Fig); we did not observe changes in induction of *Drs* or *Xbp1* splicing when SREBP was manipulated (S8D Fig).

In addition to the Ire1/Xbp1 pathway, the unfolded protein response is initiated by two other stress sensors: protein kinase R-like ER kinase (PERK, also called EIF2AK3, and named PEK in *Drosophila*) and activating transcription factor 6 (Atf6) [47]. We asked whether these other arms of the unfolded protein response played a role in the induction of the Kennedy pathway by Toll signaling. Toll signaling in fat body leads to a significant reduction in fat body *PEK* mRNA levels, and we find that loss of *PEK* does not lead to significant changes in transcript levels of *Drs*, *eas*, or *Pcyt1* in animals with active Toll signaling in fat body (S9A Fig). In contrast, *Atf6* expression is modestly increased in response to fat body Toll signaling (S9B Fig). While Toll^10b^ induces a 2.5-fold increase in *eas* compared with control fat bodies, loss of *Atf6* blunts this effect of Toll, leading to only a 1.5-fold increase in *eas* expression relative to fat bodies with *Atf6* knockdown alone. Effects of Atf6 on *Pcyt1* expression were difficult to interpret due to variance in expression of this enzyme in fat bodies expressing Atf6^RNAi^ (S9B Fig). Together, these results suggest that Toll signaling in the fat body induces two arms of the unfolded protein response: Xbp1, downstream of Ire1, and Atf6, and that Xbp1 participates in both basal and stimulated expression of Kennedy pathway enzymes.

### Toll signaling leads to ER expansion

**Fig 6:**
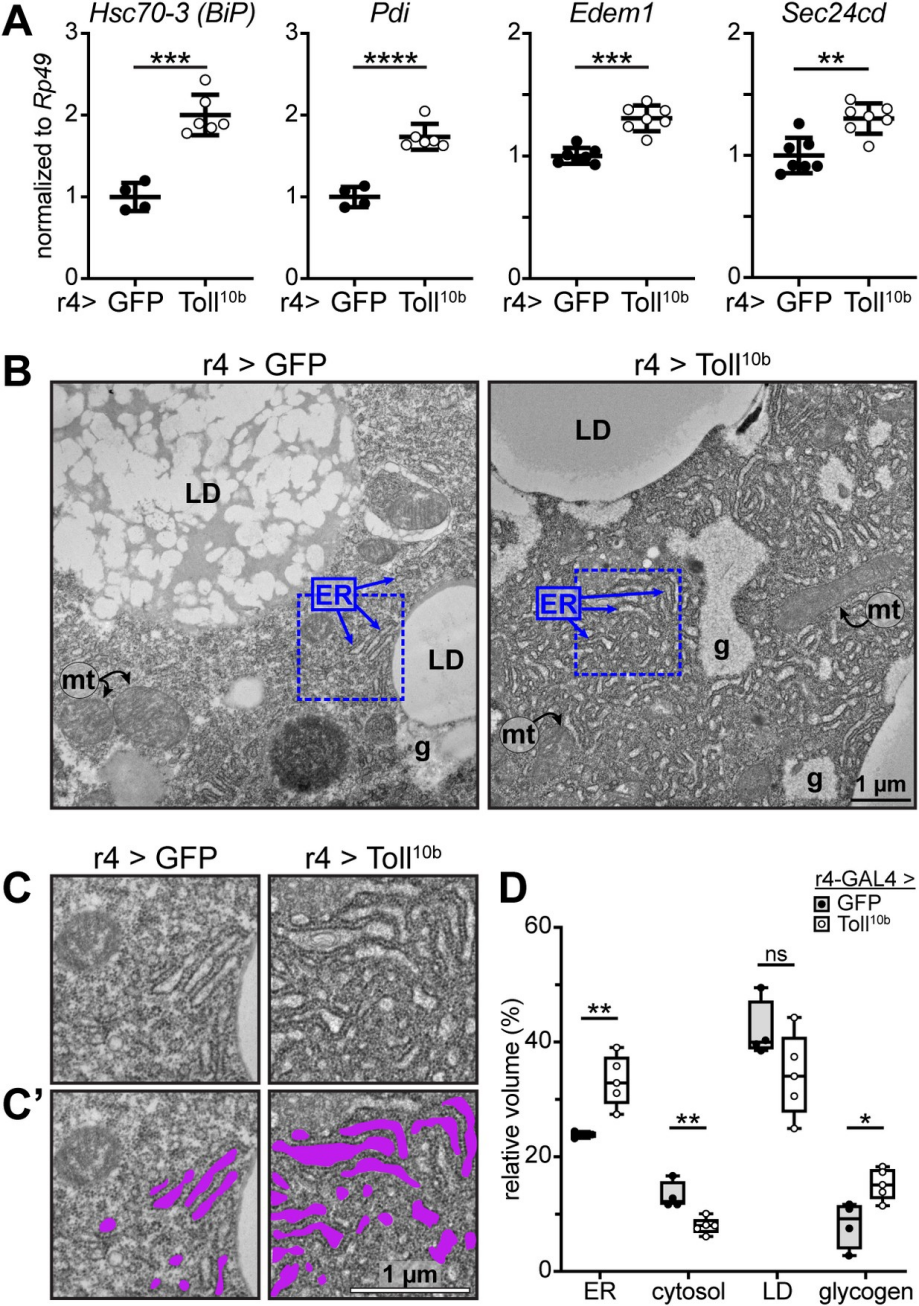
Chronic fat body Toll signaling leads to expansion of the endoplasmic reticulum. (**A**) Late third instar fat body levels of *Hsc70-3 (BiP)*, *Pdi*, *Edem1*, and *Sec24cd* were measured by RT-qPCR and normalized to *Rp49*, n = 4-8/group. **p = 0.0012, ***p = 0.001, and ****p < 0.0001 versus GFP. (**B**) Electron micrographs of fat body cells expressing GFP (left) or Toll^10b^ (right) under control of r4-GAL4. ER, endoplasmic reticulum; LD, lipid droplet; g, glycogen; mt, mitochondria. Dashed blue lines show areas enlarged in (C and C’). Scale bar, 1 μm. (**C**) Insets of electron micrographs outlined in blue dashed boxes in (B). (**C’**) Insets with ER structures highlighted in purple. Scale bar, 1μm. (**D**) Stereological analysis of organelle- and feature-specific volume density. Data points represent the average of volume densities measured from 5–15 images from a single fat body, n = 4–5 fat bodies/group. *p = 0.0175 and **p ≤ 0.0044 versus GFP. Data are presented as means ± SD. p values were determined by Student’s t test (A,D).

The induction of *Xbp1* splicing suggested that the ER unfolded protein response might be induced in fat bodies with active innate immune signaling. We therefore examined expression of canonical Xbp1 targets that participate in the unfolded protein response [48,49]. We found significantly elevated expression of *binding immunoglobulin protein* (*BiP*, encoded by *Hsc70-3*), *protein disulfide isomerase* (*Pdi*), *ER degradation enhancer mannosidase alpha-like 1* (*Edem1*), and *Sec24cd* in response to chronic Toll signaling (Figs 6A and S3G).

We next used transmission electron microscopy to examine whether ER morphology is altered by innate immune signaling. We observed dilated ER in fat body cells with active Toll signaling compared with controls (Fig 6B–6C’). To quantify this difference, we used stereological tools to compare organelles and features such as glycogen and cytosol between genotypes. This analysis revealed a 40% increase in the relative volume of ER with a simultaneous 40% decrease in the relative volume of organelle-free cytosol in response to Toll pathway activation. We find no significant difference in the relative volume of lipid droplets, but we observe an increase in glycogen volume in fat body cells expressing Toll^10b^ compared with controls, in agreement with our finding of increased glycogen levels in the larval fat body (Fig 6D and refer back to Fig 2B).

### AMP synthesis contributes to induction of Kennedy pathway enzymes

**Fig 7:**
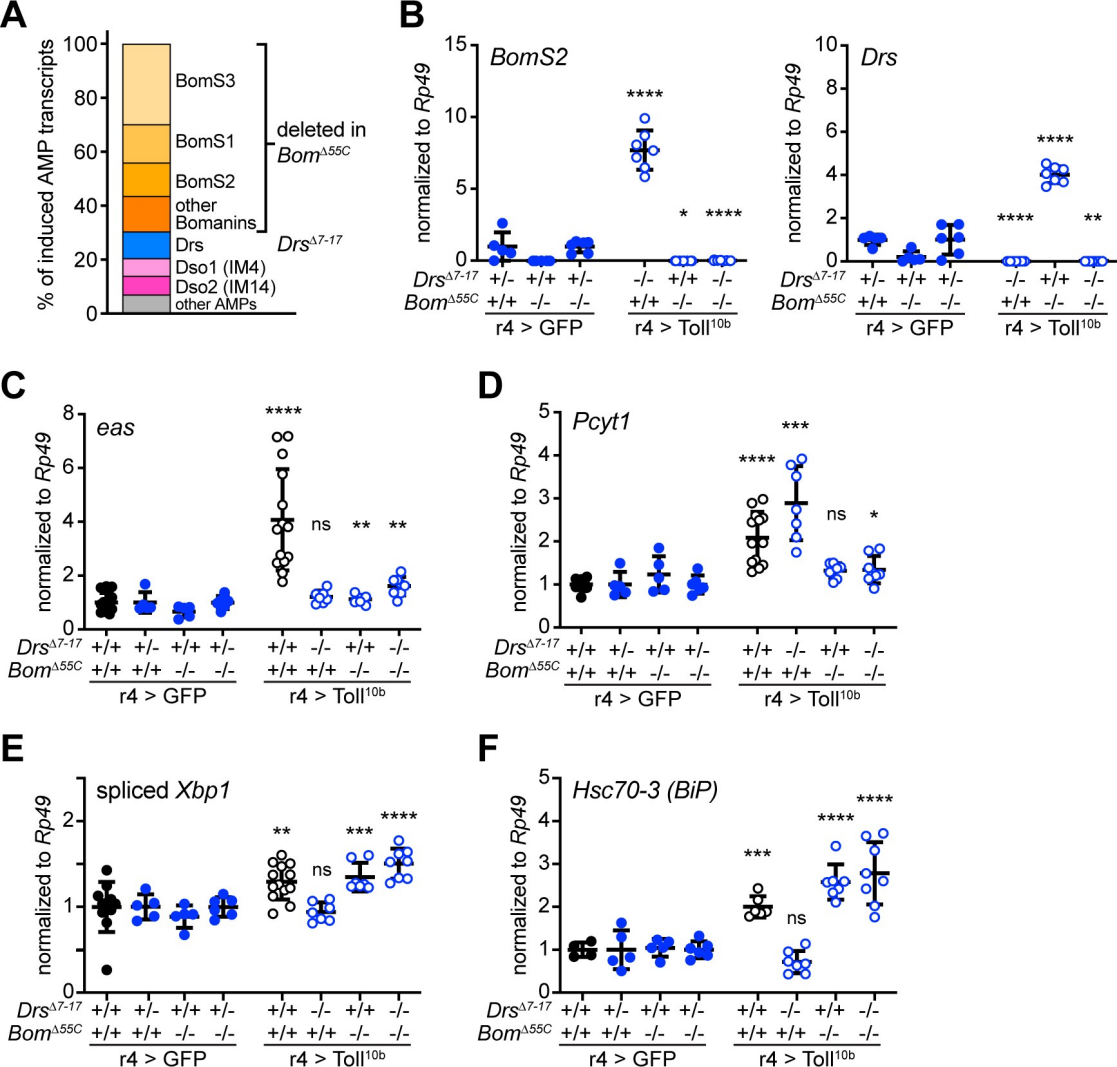
Induction of antimicrobial peptide synthesis contributes to regulation of eas and Pcyt1 by Toll signaling. (**A**) Toll signaling induces expression of 17 AMPs. AMPs clustered on chromosome 2 (deleted in the *Bom*^*Δ55C*^ mutant) represent 70% of the AMP transcripts induced by Toll^10b^ expression. Drs, deleted in the *Drs*^*Δ7–17*^ mutant, represents 10% of the induced AMP transcripts. (**B**) Transcript levels of *BomS2* (left) and *Drs* (right), normalized to *Rp49*, in fat bodies of late third instar larvae expressing GFP (closed symbols) or Toll^10b^ (open symbols) under r4-GAL4 control. Animals were wild type, heterozygous, or homozygous for *Drs*^*Δ7–17*^ and *Bom*^*Δ55C*^ as indicated, n = 5-8/group. *p = 0.0145, **p = 0.0015, and ****p < 0.0001 versus GFP-expressing controls with the same *Drs*^*Δ7–17*^ and *Bom*^*Δ55C*^ genotypes. Note that GFP-expressing controls are heterozygous for *Drs*^*Δ7–17*^ while Toll^10b^-expressing larvae are homozygous for *Drs*^*Δ7–17*^. (**C, D**) Transcript levels of *eas* (C) and *Pcyt1* (D), normalized to *Rp49*, in fat bodies of late third instar larvae expressing GFP (closed symbols) or Toll^10b^ (open symbols) under r4-GAL4 control. Animals were wild type, heterozygous, or homozygous for *Drs*^*Δ7–17*^ and *Bom*^*Δ55C*^ as indicated, n = 5-8/group (blue open and closed symbols) and n = 11-14/group for animals expressing GFP or Toll^10b^ in fat body on a wild type background (black open and closed symbols). *p ≤ 0.0409, **p ≤ 0.032, ***p = 0.0009, and ****p < 0.0001. (**E, F**) Transcript levels of spliced *Xbp1* (E) and *Hsc70-3 (BiP)* (F), normalized to *Rp49*, in fat bodies of late third instar larvae expressing GFP (closed symbols) or Toll^10b^ (open symbols) under r4-GAL4 control. Animals were wild type, heterozygous, or homozygous for *Drs*^*Δ7–17*^ and *Bom*^*Δ55C*^ as indicated, n = 5-8/group (blue open and closed symbols), n = 11-13/group (spliced *Xbp1*), and n = 4-6/group (*Hsc70-3* (*BiP*)) for animals expressing GFP or Toll^10b^ in fat body on a wild type background (black open and closed symbols). **p = 0.0086, ***p = 0.0004, and ****p < 0.0001. Data are presented as means ± SD. p values were determined by Student’s t test.

In response to infection with fungi or Gram-positive bacteria, the Toll signaling pathway drives synthesis and secretion of large quantities of AMPs via the classical ER-Golgi-secretory vesicle pathway [50]. Individual AMPs have been measured at levels close to 100 μM in hemolymph, which represents secretion of millions of individual proteins [16]. Acute activation of Toll signaling leads to 4- to 410-fold induction of 17 of the 37 AMPs encoded in the *Drosophila* genome (S10A Fig) [26]. We reasoned that ER expansion and phospholipid synthesis may serve the process of AMP production and secretion. To test this hypothesis, we asked whether induction of Kennedy pathway enzymes occurs downstream of AMP synthesis. The *Bom*^*Δ55C*^ mutation deletes a cluster of ten highly-induced genes, the Bomanins, that are critical for survival during infection [51], and the *Drs*^*Δ7–17*^ mutation is a deletion of *Drosomycin* [28]. Together, the genes encoded in the *Bom*^*55C*^ cluster and *Drs* account for 80% of the AMP transcripts that are induced by Toll signaling (Fig 7A). In a series of genetic manipulations to curtail AMP production, we drove Toll^10b^ in fat bodies of larvae carrying *Bom*^*Δ55C*^ and *Drs*^*Δ7–17*^ mutations in single or double homozygous combinations and measured expression of the Kennedy pathway enzymes *eas* and *Pcyt1*. The *Bom*^*Δ55C*^ and *Drs*^*Δ7–17*^ mutations, alone or in combination, led to the expected decreases in Toll^10b^-induced expression of the *Bom*^*55C*^ gene *BomS2* and *Drs* (Fig 7B). Unmanipulated AMPs such as *Daisho1* and *Daisho2* (also known as *IM4* and *IM14*) [52] were still induced by Toll^10b^ in *Bom*^*Δ55C*^ and *Drs*^*Δ7–17*^ single or double mutants (S10B Fig). In larval fat bodies expressing GFP, mutation of *Bom*^*55C*^ and *Drs* did not alter low, basal levels of *eas* or *Pcyt1*. However, *Bom*^*Δ55C*^*; Drs*^*Δ7–17*^ double homozygotes expressing Toll^10b^ exhibited only a 1.6-fold induction of *eas* and a 1.3-fold induction of *Pcyt1*. In contrast, in larvae with a full complement of AMPs, Toll signaling elicited a 4.1-fold induction of *eas* and a 2.1-fold induction of *Pcyt1* (Fig 7C and 7D). These data suggest that induction of Kennedy pathway enzymes in response to Toll signaling is closely linked to the level of AMP synthesis.

Next, we asked whether ER stress genes were also suppressed in *Bom*^*Δ55C*^*; Drs*^*Δ7–17*^ double homozygous fat bodies. Surprisingly, transcript levels of spliced *Xbp1* and *Hsc70-3* (*BiP*) were increased in these fat bodies compared with controls and were elevated above levels observed in animals expressing Toll^10b^ with a full complement of AMP genes (Fig 7E and 7F). These data challenged our previous findings that Xbp1 positively contributes to regulation of *Pcyt1* and *eas* downstream of Toll receptor activation. This result led us to consider why ER stress might be induced in cells lacking a substantial portion of the AMP genes that would contribute to the secretory burden induced by Toll.

We surmised that fat bodies undergoing immune signaling but with impaired induction of Pcyt1 and eas might exhibit a relative lack of capacity for phospholipid synthesis. Because changes in phospholipid synthesis can induce ER stress [53,54], we measured expression of ER stress genes in fat bodies with simultaneous knockdown of Pcyt1 and eas. Expression of RNAi transgenes targeting these enzymes strongly reduced their levels in fat bodies (S11A Fig). As we saw previously, expression of Toll^10b^ in fat body led to increased protein levels of both Pcyt1 and eas, and co-expression of Toll^10b^ with RNAi transgenes targeting these enzymes strongly reduced Pcyt1 expression and normalized eas to control levels (S11A Fig). We note that reduced Pcyt1 and eas expression in fat body did not restore triglyceride levels in animals co-expressing Toll^10b^ in fat body (S11B Fig). We measured transcript levels of spliced *Xbp1* and its targets *Hsc70-3* (*BiP*), *Pdi*, and *Sec24cd* in fat bodies expressing control transgenes or Pcyt1^RNAi^ and eas^RNAi^ with or without co-expression of Toll^l0b^. We find that, indeed, loss of these Kennedy pathway enzymes induces *Xbp1* splicing and expression of *Hsc70-3* (*BiP*) and *Sec24cd* in control fat bodies and those expressing Toll^10b^ (S11C Fig).

### Pcyt1 and easily shocked support the innate immune response

**Fig 8:**
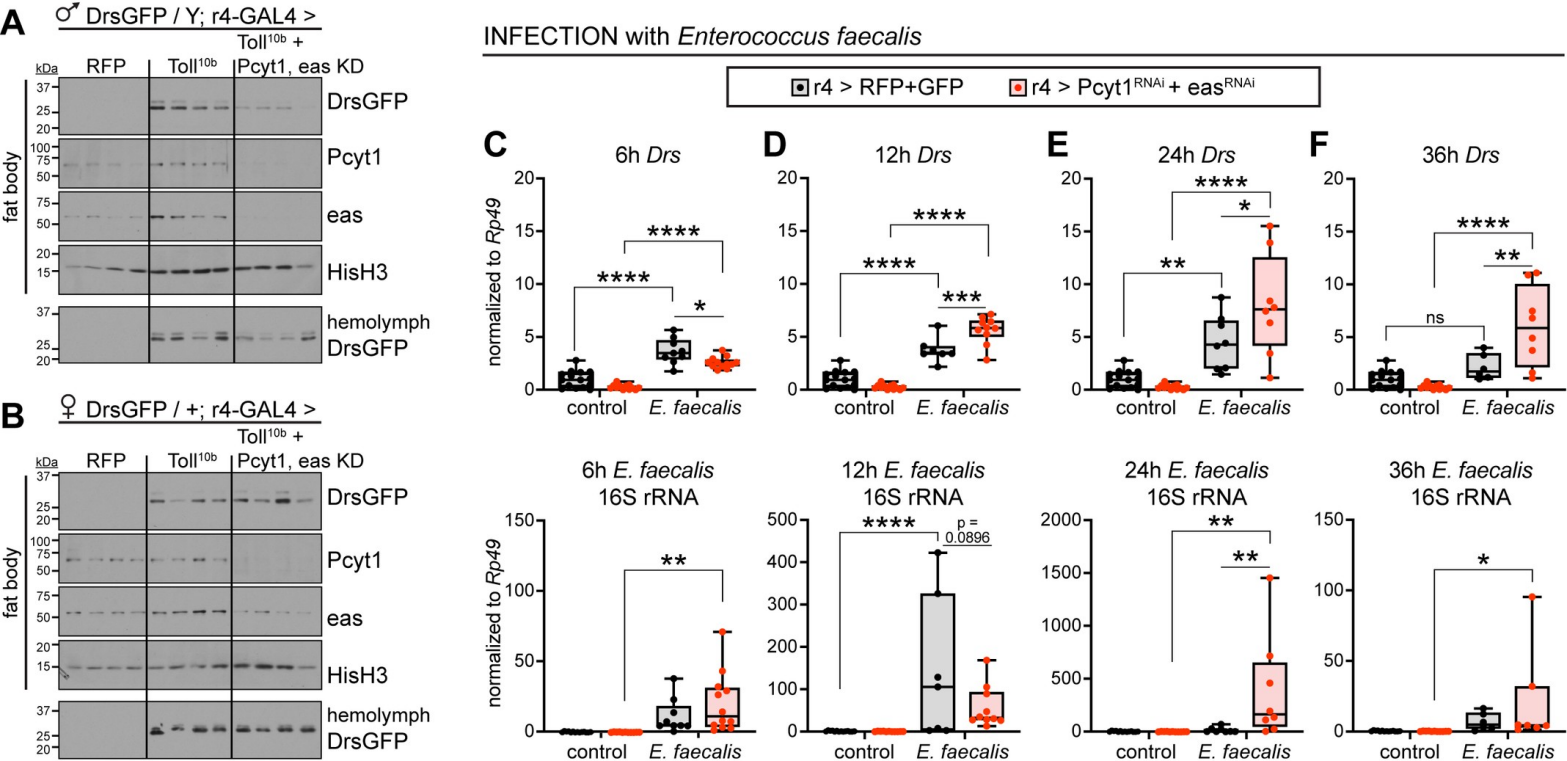
Pcyt1 and easily shocked support the innate immune response. (**A, B**) Western blot analysis of DrsGFP, Pcyt1, and eas protein levels in fat bodies (top four panels) and DrsGFP in hemolymph (bottom panel) from male (A) and female (B) late third instar larvae expressing RFP or Toll^10b^ alone or with simultaneous knockdown of Pcyt1 and easily shocked using r4-GAL4. Histone H3 levels are shown as loading controls for fat body blots. For hemolymph blots, equivalent volumes of diluted hemolymph (equal to 50 nL of neat hemolymph) were loaded. (**C-F**) Top: Whole-animal transcript levels of *Drs*, and bottom: transcript levels of *Enterococcus faecalis* 16S rRNA in larvae expressing RFP+GFP or Pcyt1^RNAi^+eas^RNAi^ in fat body under control of r4-GAL4 at 6–36 hours post infection with *Enterococcus faecalis*. Both *Drs* and *Enterococcus faecalis* 16S rRNA were normalized to *Rp49*. Uninfected control samples for each genotype were collected throughout the time course, pooled, and are plotted on each graph. All groups were normalized to the pooled, uninfected RFP+GFP samples, n = 6-15/group. *p ≤ 0.0310, **p ≤ 0.0097, ***p ≤ 0.0007, and ****p < 0.0001 versus infected RFP+GFP or versus uninfected controls of the same genotype, as indicated. Data are presented as means ± SD. p values were determined by one-way ANOVA with the Tukey-Kramer multiple comparisons test (C-F).

To determine whether Pcyt1 and eas play a functional role in the immune response, we assessed two immune functions, AMP production and bacterial clearance, in larvae with simultaneous knockdown of Pcyt1 and eas in fat body. First, we genetically activated the Toll signaling pathway by expressing Toll^10b^ in larval fat bodies. To evaluate AMP secretion, we used a DrsGFP transgene that is a fusion protein of Drosomycin and GFP under the control of 2.5 kb of the *Drosomycin* promoter upstream of the start codon. This DrsGFP construct retains the signal peptide necessary for AMP secretion into hemolymph. This allowed us to assess secretion of Drosomycin from the fat body into hemolymph during active Toll signaling. In male flies expressing Toll^10b^ in fat body, we observed increased protein levels of DrsGFP, Pcyt1, and eas in the fat body. Knockdown of Pcyt1 and eas in fat body led to a strong, tissue-autonomous decrease in DrsGFP protein levels (Fig 8A). DrsGFP is undetectable in hemolymph from control larvae expressing RFP in fat body. Secretion of DrsGFP into hemolymph is markedly increased by fat body Toll signaling, but this is reduced when Pcyt1 and eas are knocked down (Fig 8A). In female larvae, knockdown of Pcyt1 and eas in fat bodies with active Toll signaling did not have an appreciable effect on DrsGFP expression in fat body or secretion into hemolymph, raising the possibility that the humoral immune response operates differently in males and females (Fig 8B).

Finally, we investigated the role of Pcyt1 and eas in the ability of the animal to elicit an appropriate immune response via induction of *Drosomycin* and to properly clear the bacterial insult. We infected larvae with *Enterococcus faecalis* and measured *Drs* mRNA and *E*. *faecalis* 16S rRNA in the same samples of total RNA isolated from whole larvae at various time points following infection. By 6 hours post infection, animals expressing RFP and GFP control transgenes in fat body and animals with simultaneous knockdown of Pcyt1 and eas have induced *Drs* gene expression, although *Drs* mRNA levels in the knockdown animals were significantly lower compared with RFP and GFP controls. At this time point we observed 16-54-fold higher levels of *E*. *faecalis* 16S rRNA in infected animals compared with uninfected controls (Fig 8C). We note that *E*. *faecalis* is a minor component of the *Drosophila* microbiome [55], and low levels of 16S rRNA were detected in controls of both genotypes. By 12 hours post infection, the knockdown animals succeeded in inducing *Drs* transcripts and matched controls in the amount of *E*. *faecalis* bacterial transcripts (Fig 8D). Thus, at early time points of infection, knockdown animals lag in their ability to induce *Drs* compared with control animals. At 24 hours post infection, infected animals with fat body knockdown of Pcyt1 and eas exhibited significantly increased *Drs* levels compared with infected controls. This difference correlates with 22-fold higher mean levels of bacterial 16S rRNA in the infected knockdown animals compared with infected controls (Fig 8E). By 36 hours post infection, *Drs* levels in control animals expressing RFP and GFP in fat body were equivalent in uninfected and infected groups. In contrast, infected animals with loss of Pcyt1 and eas in fat body sustained an increase in *Drs* levels (Fig 8F). Mean values of *E*. *faecalis* 16S rRNA were 56-fold higher in infected knockdown animals compared with infected controls expressing RFP and GFP, although this difference is not statistically significant (Fig 8F). We note that *Drs* levels at 24 and 36 hours post infection correlate with bacterial load, suggesting that, at these late time points, *Drs* expression is driven by persistence of bacteria in the animal, rather than from the initial stress of injury. These data suggest that the metabolic switch to phospholipid synthesis in the fat body in response to immune signaling may contribute to the ability of the animal to control the microbial burden during an infection.

## Discussion

Here we show that the Toll pathway acts in a tissue-autonomous manner to reduce triglyceride storage and increase phospholipid levels in the *Drosophila* larval fat body, an organ that carries out the humoral functions of the innate immune system. Transgenic expression of a constitutively-active Toll^10b^ transgene or physiological activation of Toll signaling by cuticular wound or bacterial infection leads to decreased triglyceride storage and reduced expression of the DGAT homolog *midway*, an enzyme that regulates the final step of triglyceride synthesis. A serious long-term consequence of decreased nutrient storage is increased sensitivity to desiccation, a phenotype caused by impaired larval triglyceride levels. While fat bodies with active Toll signaling have reduced triglycerides, they have increased levels of major PE and PC species. Transcript levels of Kennedy pathway enzymes that synthesize PE and PC are increased in larvae with genetically or physiologically activated Toll signaling in fat body. The transcription factor Xbp1, an important regulator of ER biogenesis and the unfolded protein response, contributes to the induction of phospholipid enzyme transcripts in response to Toll^10b^ expression in fat body. Accordingly, morphological analysis shows expanded and dilated ER in fat bodies with active Toll signaling. In response to activation of the Toll pathway, the fat body synthesizes and secretes massive quantities of AMPs into hemolymph to defend against pathogenic Gram-positive bacteria and fungi. We show that deletion of AMP genes to reduce this secretory load blunts the Toll-dependent induction of Kennedy pathway enzymes, yet we still find activation of *Xbp1* in AMP mutants, indicating a complex relationship between ER stress and the transcriptional regulation of phospholipid synthesis downstream of Toll signaling. An immediate consequence of shifting larval fat body lipid metabolism toward phospholipid synthesis is to support the immune response; Pcyt1 and eas act in the fat body to allow the larva to induce AMP genes at early time points and to help the animal clear bacterial infection at later time points, likely by supporting and sustaining AMP production at a high level.

The synthesis of phospholipids and triglycerides is highly dynamic both over development and between different tissue types in *Drosophila* [38]. Our results demonstrate a reciprocal relationship between triglyceride and phospholipid levels in the *Drosophila* larval fat body that is dependent on the physiological context of the organ. Activating innate immune signaling in larval fat body leads to a decrease in triglyceride storage and increases in PE and PC species. It will be of interest to determine whether the changes we observe in the larval fat body also occur in adult flies, which unlike larvae, are not actively growing. A tradeoff between triglyceride storage and membrane phospholipid levels is observed in other contexts. In S2 cells, in adult flies, in *C*. *elegans*, and in mammals, loss of Kennedy pathway enzymes leads to elevated triglyceride levels [56–60]. In contrast, manipulations that reduce lipin and DGAT activity directly or indirectly by disrupting Torsin function lead to elevated phospholipid levels and reduced triglyceride synthesis [35,61,62]. These studies and ours suggest that cells tightly regulate the balance between stored neutral lipids and membrane phospholipids.

Reduced triglyceride storage in animals with genetic or physiological activation of fat body Toll signaling may be due, at least in part, to changes in expression of two key lipogenic enzymes, the phosphatidic acid phosphatase Lipin and the DGAT homolog midway. Indeed, we were able to partially rescue impaired triglyceride storage caused by Toll signaling by forcing simultaneous expression of Lipin and midway in fat body. A possible explanation for the failure of Lipin and midway to fully rescue triglyceride storage is that Toll signaling may regulate Lipin or midway not only at the level of gene expression but also at the level of enzyme activity and/or subcellular localization. Lipin is mainly regulated post-translationally, with phosphorylation altering both enzymatic activity and subcellular localization [63]. Our data show a small but significant decrease in Lipin activity in fat bodies expressing Toll^10b^. However, while forced expression of Lipin substantially increases total Lipin activity in fat bodies with active Toll signaling, this increase in activity does not lead to increased triglyceride storage. These data are compatible with a model in which Toll signaling disrupts subcellular localization of Lipin such that transgenic expression of either Lipin or midway would be insufficient to promote wild type levels of triglyceride accumulation. For example, flies with loss of the ER membrane protein Torsin exhibit an increase in nuclear Lipin and elevated triglyceride levels [35], and in mammals, insulin signaling promotes lipin activity by changing its subcellular distribution [64]. Our previous work shows that Toll signaling in fat body inhibits insulin signaling and that restoring insulin signaling rescues triglyceride storage [25]. Therefore, Toll signaling might be expected to dominantly inhibit Lipin function by altering its subcellular localization. Finally, the failure of forced Lipin and midway expression to fully restore triglyceride accumulation in fat bodies with active Toll signaling may be due to an increase in flux of lipid intermediates into the phospholipid synthesis pathway.

Major species of the membrane phospholipids PE and PC accumulate in response to Toll pathway activation. Our results suggest that increased phospholipid levels are likely due to increased *de novo* synthesis of PE and PC because transcripts encoding Kennedy pathway enzymes are increased in fat bodies expressing Toll^10b^ and, in the case of Pcyt1, in response to infection. We also find elevated protein levels of eas and Pcyt1, enzymes that carry out rate-limiting first and second steps, respectively, in the PE and PC synthesis pathways [65,66] in fat bodies expressing Toll^10b^. Our data as a whole suggest that PE and PC may be involved in the secretory function of the fat body during the immune response.

Our investigation into the transcriptional mechanism underlying increased expression of genes encoding Kennedy pathway enzymes led us to discover that Xbp1 participates in this aspect of the immune response in *Drosophila*. Xbp1 is a transcription factor that is activated by splicing of its transcript when high secretory demand leads to an abundance of misfolded proteins in the ER. Xbp1 serves as an essential mediator of the unfolded protein response that relieves ER stress, and its role in the whole animal extends to supporting function of cells with high secretory capacity. For example, during differentiation of naïve B cells into immunoglobulin-secreting plasma cells, Xbp1 is induced and is required for maximal immunoglobulin secretion [11]. Additionally, *Xbp1* is required for membrane phospholipid synthesis and ER expansion that accompany plasma cell differentiation [9,10,67]. In mouse fibroblasts, forced expression of spliced *Xbp1* is sufficient to induce PC and PE synthesis via the Kennedy pathway and to drive ER biogenesis [43,68]. Our data show that levels of spliced *Xbp1* are increased in response to Toll signaling and that Xbp1 is necessary for maximal induction of *eas*, *CG7149*, and *Pcyt1* in response to acute activation of fat body Toll signaling. Activation of the humoral immune response in the fat body induces high levels of AMP synthesis, leading to a large secretory burden in these cells. Indeed, we find a 40% expansion of the ER in fat body cells with active immune signaling. We considered that the AMP secretory load might be a component of the pathway leading to induction of Kennedy pathway enzymes that synthesize ER membrane phospholipids. We find that relieving secretory demand by deletion of AMP genes blunts induction of *eas* and *Pcyt1*. Our results suggest that immune effector production leading to phospholipid synthesis and subsequent ER biogenesis that supports immune function is an ancient and conserved component of the immune response.

What is the relationship between Toll signaling, AMP production, ER stress, and induction of Kennedy pathway enzymes? Our data indicate a positive role for Xbp1 in acute induction of *Pcyt1* and *eas* in response to Toll signaling, and we find an important role for Xbp1 in basal expression of *Pcyt1*. However, one curious finding from the AMP deletion experiment is that while Toll pathway induction of *Pcyt1* and *eas* is blunted in cells lacking *Drs* and the *Bom*^*55C*^ cluster of ten AMP genes, the ER stress response is induced. We find increased levels of both spliced *Xbp1* and *Hsc70-3* (*BiP*) in *Drs*^*Δ7–17*^; *Bom*^*Δ55C*^ fat bodies with active Toll signaling. This result uncouples Kennedy pathway gene expression from Xbp1 and therefore contradicts a simple linear model in which Toll-dependent induction of AMP synthesis leads to ER stress and activates Xbp1, which, in turn, activates expression of *Pcyt1* and *eas*. Our data suggest an explanation for the paradoxical finding of elevated ER stress in cells with reduced AMP secretory burden. Because Toll^10b^-activated *Drs*^*Δ7–17*^; *Bom*^*Δ55C*^ fat body cells have lower-than-normal induction of Kennedy pathway enzymes, we reasoned that they might experience a relative phospholipid deficiency, perhaps in relation to other components of ER biogenesis that might also be activated in response to Toll signaling. In support of this idea, we find elevated levels of spliced *Xbp1* and its targets *Hsc70-3* (*BiP*) and *Sec24cd* in otherwise wild type fat body cells lacking Pcyt1 and eas. In sum, while we have not strictly tested the sufficiency of Xbp1 in inducing Kennedy pathway enzymes in the *Drosophila* larval fat body, our data showing elevated *Xbp1* splicing but relatively low levels of *Pcyt1* and *eas* in Toll^10b^-expressing *Drs*^*Δ7–17*^; *Bom*^*Δ55C*^ fat body cells indicate that there is a missing component in our understanding of how these lipid metabolic enzymes are regulated downstream of Toll and Dif. One possibility is that Pcyt1 and eas are induced in an anticipatory manner and that there is a retrograde signaling event that then titrates levels of Kennedy pathway enzyme expression to the actual secretory demand. Future work will be needed to identify the transcriptional mechanisms that activate phospholipid synthesis downstream of Toll signaling and AMP production.

The shift from nutrient storage to membrane phospholipid synthesis induced by Toll signaling likely has both immediate and long-term consequences for animal survival. The lipid metabolic switch accompanies ER expansion, and biogenesis of ER is predicted to sustain secretion of AMPs during infection. On the other hand, it is critical to tightly regulate ER function during infection. For example, unrestrained Ire1 activity and Xbp1 splicing in *sigma-1 receptor* knockout mice leads to elevated proinflammatory cytokine production and increased rates of sepsis in response to LPS treatment [69]. A clear disadvantage of shifting fatty acids from nutrient storage to phospholipid synthesis in response to Toll signaling in flies is the large decrease in stored energy available for metamorphosis and early adult life. Animals with active Toll signaling in fat body enter the pupal stage with 50% less triglyceride compared with control animals. Impaired triglyceride storage in the larval stage may reduce metabolic energy available to complete metamorphosis and decrease the pool of triglyceride that is reserved for waterproofing the adult cuticle to protect against desiccation [31]. Adult flies that experienced active Toll signaling in the larval and pupal fat body are highly sensitive to desiccation stress, suggesting that the failure to store triglycerides in the larval stage leads to prolonged disruptions to homeostasis and stress resistance.

Our data suggest that induction of the Kennedy pathway contributes to the immune response in *Drosophila*. Our results expand the range of processes in which Kennedy pathway enzymes participate. Loss-of-function mutations in *eas* and *Pcyt1* lead to reduced levels of PE and PC [70,71], and these mutations lead to defects in neuronal morphogenesis and excitability [72,73], oocyte development [74], and cardiac function [59]. We find that knockdown of Pcyt1 and eas leads to reduced secretion of at least one antimicrobial peptide, Drs, into hemolymph in male larvae with active fat body Toll signaling. Importantly, loss of these two metabolic enzymes disrupts the response to an immune challenge with the bacteria *Enterococcus faecalis*. Larvae lacking expression of Pcyt1 and eas in fat body exhibit impaired AMP expression during the acute phase of infection and experience greater bacterial burden at late time points of infection compared with wild type animals. These data suggest that phospholipid synthesis supports secretory function during the innate immune response.

Taken together, our results show that Toll signaling leads to a profound change in lipid metabolism that supports immune function but also hinders the ability of the animal to store energy. Our work raises the question of how animals balance the metabolic demands of infection with the capacity to survive periods of reduced nutrient availability. Furthermore, it will be of interest to determine whether chronic changes in lipid metabolism induced by innate immune signaling underlie pathologies of inflammatory diseases.

## Materials and methods

### *Drosophila* stocks and husbandry

Flies were raised on food containing 7.8% molasses, 2.4% yeast, 4.6% cornmeal, 0.3% propionic acid, and 0.1% methylparaben (Archon Scientific, Durham, NC). Except where noted, experiments were performed using mid- to late-third instar larvae (96–108 h after egg lay). The following stocks were obtained from the Bloomington *Drosophila* Stock Center (Bloomington, IN): UAS-RFP (2nd (#30556) and 3rd (#31417) chromosome insertions), UAS-GFP (2nd (#1521) and 3rd (#1522) chromosome insertions), UAS-Dif^RNAi^ (#30513), *mdy*^*QX25*^ (#5095), *mdy*^*EY07280*^ (#20167), UAS-midway^RNAi^ (#65963), UAS-Lipin^RNAi^ (#63614), UAS-Pcyt1^RNAi^ (#62156), UAS-Xbp1^RNAi^ (#36755), UAS-Atf6^RNAi^ (#26211), UAS-PEK^RNAi^ (#42499), DiptLacZ, DrsGFP (#55707), r4-GAL4 (#33832) and cg-GAL4 (#7011). UAS-SREBP^RNAi^ (#37640) and UAS-eas^RNAi^ (#34287) were obtained from the Vienna *Drosophila* Resource Center. Other flies used were: UAS-Toll^10b^ [75], UAS-Dif [76], DrsGFP [30], UAS-Lipin [36], *Bom*^*Δ55C*^ [51], and *Drs*^*Δ7–17*^ [28]. Full genotypes of flies are listed in S1 Table.

### Construction of UAS-HA.mdy transgenic flies

The full-length *midway* (*mdy*) cDNA was amplified by PCR from clone LD33582 (*Drosophila* Genomics Resource Center, Bloomington, IN) using gene-specific primers engineered to contain an amino-terminal HA tag (see S2 Table). PCR products were cloned into pENTR (Invitrogen) and validated by sequencing. Gateway cloning (Invitrogen) was used to generate pUAST-HA.mdy. This construct was injected into *Drosophila* embryos at Rainbow Transgenics (Camarillo, CA). Standard genetics was used to map and generate balanced transgenic lines.

### Bacterial infection

Early third instar larvae (~72–78 h after egg lay) were removed from bottles, rinsed in PBS, sorted for fluorescent transgene expression as indicated, and transferred to fresh food. For bacterial infections, larvae were transferred briefly to a pool of the Gram-positive bacteria *Enterococcus faecalis* (strain OG1RF, ATCC 47077), grown in brain heart infusion media with rifampicin, and concentrated to OD_600_ 10.0 in PBS. Larvae coated in *E*. *faecalis* were then transferred to a 50 μL pool of mineral oil (M5904, Sigma-Aldrich) on a stack of eight parafilm sheets. Punctures were performed in mineral oil, which provided sufficient hydrostatic pressure to prevent the gut and fat body from bursting out of the wound site, as regularly occurs when punctures are performed in PBS. For bacterial infections and sterile injury, larvae were punctured laterally in abdominal segment 7 or 8 with a tungsten carbide needle with a one μm tip (10130–10, Fine Science Tools) [26,77]. Larvae in the sterile injury groups were transferred directly to mineral oil and punctured, and control larvae were transferred directly to mineral oil. Larvae in all three groups were kept in mineral oil on ice for ten minutes before transfer to fresh food for indicated times. Uninfected controls and sterile injury or bacteria-infected larvae bearing melanotic clots at puncture sites were collected individually for measurement of triglyceride levels and DrsGFP expression. Fat bodies dissected from two animals per sample or single, whole larvae were used for RNA isolation.

### Triglyceride and protein measurements

Whole larvae or dissected organs were flash frozen on dry ice. Samples were sonicated three times for 10 seconds each time in 140 mM NaCl, 50 mM Tris-HCl, pH 7.4, 0.1% Triton X-100 with protease inhibitors (Roche). Following clearing by centrifugation at 4°C, supernatants were transferred to new tubes. Triglyceride (Liquicolor Test, Stanbio) and protein (BCA assay, Pierce) were measured in each sample, and triglyceride levels were normalized to protein levels.

### Hemolymph trehalose and glucose

Hemolymph was collected on ice from mid-third instar larvae (hemolymph from 8–10 larvae pooled per sample). Endogenous trehalase was destroyed by heating hemolymph diluted in PBS at 70°C for 20 min. The sample was split in half, 1 mU trehalase (Sigma-Aldrich, T8778) was added to one tube, and both were incubated at 37°C for 2 h. Glucose was measured in both samples (GAGO20 kit, Sigma-Aldrich). Trehalose was calculated by subtracting glucose values in trehalase-free samples from glucose values in trehalase-treated samples, and then dividing by two as trehalose is a dimer of glucose. Trehalose and glucose values were normalized to hemolymph volume.

### Glycogen and free glucose measurements

Whole larvae or dissected organs were flash frozen on dry ice. Samples were homogenized using a Kontes pestle in 0.1 M NaOH. Following clearing by centrifugation at 4°C, samples were incubated for 1h at 37°C with 0.2 M NaOAc, pH 4.8 with or without 5 mg/mL amyloglucosidase (Sigma-Aldrich, A7420). Samples were then incubated for 10 min at room temperature with assay buffer including glucose oxidase (0.25 U/mL, Sigma-Aldrich, G7141), horseradish peroxidase (0.17 U/mL, Sigma-Aldrich, P8250), and Amplex Red (20 μM, Invitrogen, A36006). Fluorescence was measured (excitation/emission maxima = 535/587 nm) using an Infinite 200 PRO plate reader (Tecan). Glycogen was calculated as the amount of amyloglucosidase-hydrolyzed glucose, and free glucose is the amount of glucose measured in the sample without hydrolysis via amyloglucosidase. Glycogen and free glucose levels were normalized first to glucose standards (Fisher CAS 50-99-7) and then to protein levels (BCA assay, Pierce).

### Starvation

Two day-old adult male and female flies that expressed GFP or Toll^10b^ throughout the larval stage under control of r4-GAL4 were transferred to empty plastic vials (n = 9-11/vial) that were capped with a foam stopper. For starvation with water, the foam stopper was regularly saturated with water, such that water for consumption was available on the bottom side of the stopper at all times. Throughout the day, the number of dead flies was recorded every three hours.

### Western blot analysis and antibodies

Fat bodies (n = 4–6 pooled/sample) were sonicated in lysis buffer (2% SDS, 60 mM Tris-HCl, pH 6.8) with phosphatase and protease inhibitors (Roche). For hemolymph blots, hemolymph was collected from five larvae and pooled, and dilute hemolymph (equivalent to 50 nL of neat hemolymph) was separated by SDS-PAGE. Equal amounts of fat body or whole larval protein (10–40 μg/lane, measured using a BCA assay (Pierce)) were separated by SDS-PAGE, transferred to nitrocellulose, blocked in 3% milk in 1X TBS with 0.2% Tween 20 (TBS-T), and blotted overnight at 4°C with primary antibodies diluted in 1% milk in TBS-T. Following multiple washes in TBS-T, secondary antibodies were incubated in 1% milk in TBS-T for 2h at room temperature, washed again, incubated with ECL (Pierce), and exposed to film. Antibodies used were: rabbit anti-GFP (A11122, Invitrogen), rabbit anti-human Histone H3 and rabbit anti-HA (4499 and 3724, Cell Signaling Technology), rabbit anti-*Drosophila* easily shocked [78], guinea pig anti-*Drosophila* Pcyt1 [35], mouse anti-human SREBP1 (SC-13551, Santa Cruz Biotechnology), rabbit anti-GFP (A11122, Invitrogen), goat anti-rabbit HRP and goat anti-mouse HRP (111-035-003 and 115-035-003, Jackson ImmunoResearch), and goat anti-guinea pig HRP (6090–05, SouthernBiotech).

### Quantitative RT-PCR

Total RNA was extracted from whole larvae or mid to late third instar fat bodies (n = 2–6 pooled/sample) using a Direct-zol RNA MicroPrep kit (Zymo Research). DNAse-treated total RNA (1 μg) was used to generate cDNA using a High-Capacity cDNA Reverse Transcription kit (Thermo Fisher Scientific). Gene expression was measured using gene-specific primers. Quantitative PCR reactions were performed on 10–20 ng cDNA using SYBR Select Master Mix (Thermo Fisher Scientific) with a Bio-Rad CFX Connect Real-Time PCR Detection System. Relative amounts of transcripts were calculated using the comparative Ct method with *Rp49* as a reference gene [79]. Gene-specific primer sequences are listed in S2 Table.

### Lipin activity

Lipin activity was measured using Triton X-100 micelles as previously reported [80]. Briefly, radiolabeled [^32^P]PA substrate was prepared by phosphorylating 1,2-Dioleoyl-*sn*-glycerol (800811, Avanti Polar Lipids) with *E*. *coli* diacylglycerol kinase (D3065, Sigma-Aldrich) and [γ-^32^P]ATP (NEG035C005MC, Perkin Elmer) and purified by thin-layer chromatography [81]. To prepare the micelles, Triton X-100 was mixed with buffer A (50 mM Tris-HCl, 10 mM 2-mercaptoethanol, pH 7.4) to a final concentration of 10 mM. Next, 1 μmol of unlabeled 1,2-Dioleoyl-*sn*-glycero-3-phosphate (840875, Avanti Polar Lipids) was dissolved in chloroform and mixed with [^32^P]PA (3,000 cpm/nmol) in a glass tube, dried to a thin film under N_2_ gas, and resuspended with 1 mL of 10 mM Triton X-100. The measurement of PAP activity was determined by following the release of the radiolabeled phosphate from [^32^P]PA. Lysates prepared from larval fat bodies, radioactive micelles, and buffer A were combined to a final volume of 100 μL. The final concentrations for all reactions were as follows: 50 mM Tris-HCl, 10 mM 2-mercaptoethanol, and 0.2 mM PA. Total PAP activity was measured by including 0.5 mM MgCl_2_. PAP activity for Mg^2+^-independent enzymes was measured by instead including 1 mM EDTA. The reactions were allowed to proceed for 20 min at 30°C with gentle agitation and were terminated with the addition of 500 μL of acidified methanol (MeOH·0.1 N HCl). Free phosphate was extracted with the addition of 1 mL chloroform followed by 1 mL 1 M MgCl_2_. The organic extraction was vortexed and 500 μL of the aqueous phase was transferred to a scintillation vial to measure the removal of ^32^P from PA using a scintillation counter. The activity from assays containing lysate was normalized to activity in assays without enzyme present. PAP activity specific to Lipin was calculated by subtracting the Mg^2+^-independent activity from the total PAP activity and normalizing to total protein levels in each lysate.

### Thin layer chromatography

Late third instar larval fat bodies (n = 8 pooled/sample) were sonicated in 1X TBS. Fat body lysates underwent lipid extraction using a modified Bligh-Dyer method (chloroform: methanol: 0.2 M NaCl, ratio 2:2:1) [82]. Organic extracts were dried and resuspended in 30 μL of chloroform: methanol (1:1) for quantitation by thin layer chromatography. Using a Hamilton syringe (#701), 15–20 μL of resuspended extracts were spotted on the bottoms of silica gel plates (Millipore HPTLC Silica Gel Glass plates 105631) at starting points drawn in pencil. Standards were phosphatidylcholine (3 μL at 1 μg/μL; egg L-α-phosphatidylcholine Avanti Polar Lipids, 131601) and phosphatidylethanolamine (3 μL at 1 μg/μL; egg L-α-phosphatidylethanolamine Avanti Polar Lipids, 840021). During loading, samples were spotted beneath an argon gas flow to allow for rapid drying and to minimize oxidation. Silica gel plates were developed in a glass TLC tank in chloroform: methanol: acetic acid: water in a ratio of 75:35:6:2, removed, air dried, and stained overnight with iodine vapor in a separate glass TLC tank maintained in a fume hood. Following staining, plates were immediately scanned, and images were used for densitometry analysis in ImageJ to measure the area of each PE and PC spot, recognized based on the location and migration pattern of the corresponding standard. Areas of lipid spots were normalized to total protein in lysates (measured by BCA assay, Pierce) and volumes of lysates used in the initial organic extraction.

### Liquid chromatography and mass spectrometry

Larval fat bodies (n = 6 pooled/sample) were sonicated in ultrapure water and protein was measured (BCA assay, Pierce). Fat body lysates (normalized to 40 μg protein) underwent lipid extraction using a modified Bligh-Dyer method (chloroform: methanol: ultrapure water, ratio 2:2:1), adding 5 μg 1,2-dinonadecanoyl-*sn*-glycero-3-phosphocholine (DNPC, Avanti Polar Lipids, 850320) to control for extraction efficiency. Organic extracts were dried and resuspended in chloroform: methanol (1:1) for quantification by liquid chromatography-coupled mass spectrometry. Lipids were separated on an EVO C18 column (Kinetex 5 μm, 100 x 4.6 mm, Phenomenex), using a binary gradient consisting of Solvent A (69% methanol, 31% water with 10 mM ammonium acetate) and Solvent B (50% methanol, 50% isopropanol with 10 mM ammonium acetate) as the mobile phases. Phosphatidylcholine (PC) and phosphatidylethanolamine (PE) species identification and relative quantitation was achieved by liquid chromatography-linked electrospray ionization mass spectrometry on a 4000 QTRAP triple-quadrupole mass spectrometer (AB Sciex). Identification of phospholipids of interest, corresponding to PC and PE species with acyl chain lengths previously described to be abundant in larval fat body [38–40], was performed in positive ion mode via multiple reaction monitoring [83].

### Electron Microscopy

Larval fat bodies were dissected and fixed initially in 2% glutaraldehyde, 2.5% formaldehyde, 0.1 M Na cacodylate, pH 7.4 (30 min at room temperature followed by 1h on ice). They were then post-fixed in 1% OsO_4_, 0.1 M Na cacodylate, pH 7.4 (15 min on ice and 45 min at room temperature), washed three times for 5 min in 0.1 M Na acetate, pH 6.0, and then stained in-block overnight at room temperature in 0.5% uranyl acetate in the same buffer. The samples were then dehydrated in acetone (70, 95, and 100%), exchanged from acetone into Epon resin, and embedded in fresh Epon. 70 nm sections from embedded fat bodies were mounted on copper grids, stained sequentially with uranyl acetate and lead citrate, and examined by transmission electron microscopy.

### Stereology

All images for stereology were taken in an unbiased manner at 6000X. Three different blocks of each genotype (r4>GFP or r4>Toll^10b^) that each contained fat bodies from three to four larvae were used for quantitative analysis. Biological replicates refer to sets of adjacent sections cut from separate blocks or from different depths of the same block to expose different fat body tissue. STEPanizer software (Java) was used for stereology analysis [84]. In order to measure relative volumes of features within sections, images were analyzed using nine-line tile pairs per image [85]. Point counts were made to determine relative volume of organelles comprising the entire cytoplasmic landscape in each image. Batch mode was used to analyze images from each block for each genotype.

### Quantitation and statistical analysis

Statistical parameters including exact sample sizes (with n referring to biological replicates), data plotted (typically means ± SD), exact p values, and statistical tests used are reported in Figure Legends. Statistical analyses were performed using Graphpad Prism 8. Survival of starved flies was analyzed by the log-rank test. All other data were analyzed by Student’s t test or by one-way ANOVA with the Tukey-Kramer or Dunnett’s multiple comparisons test.

## Supporting information

S1 Fig Toll signaling in the larval fat body reduces triglyceride storage throughout the third instar. (**A**) Whole-animal triglyceride levels throughout the third instar (72–120 hours after egg lay (h AEL)) and in white prepupae (WPP) were normalized to protein, n = 10-11/group. *p ≤ 0.0459 and **p ≤ 0.0019 versus GFP. (**B**) Whole-animal triglyceride levels throughout the third instar and in white prepupae were normalized to body weight, n = 10-11/group. *p ≤ 0.0373, **p = 0.0033, and ***p = 0.0009 versus GFP. (**C**) Whole-animal protein levels throughout the third instar and in white prepupae were normalized to body weight, n = 10-11/group. *p = 0.0117 versus GFP. Data are presented as means ± SD. p values were determined by Student’s unpaired t test.
(TIF)

S2 Fig Toll signaling induces expression of antimicrobial peptides in response to infection. (**A**) Schematic representation of the Toll signaling pathway leading to AMP expression. In response to activation of Toll receptors, the IκB homolog cactus is phosphorylated and degraded, freeing Dif to translocate into the nucleus to regulate expression of canonical targets such as the antimicrobial peptide genes encoding Drosomycin and the Bomanin peptides. (**B**) Transcript levels of *Drs*, normalized to *Rp49*, in third instar larval fat bodies from uninfected controls, larvae subjected to sterile injury, and larvae infected with *Enterococcus faecalis* at 6–36 hours post infection n = 7-10/group. ***p ≤ 0.0009 and ****p < 0.0001 versus uninfected controls. Transcripts were normalized to *Rp49*. Data are presented as means ± SD. p values were determined by one-way ANOVA with Dunnett’s multiple comparisons test.
(TIF)

S3 Fig Genetic activation of Toll signaling with cg-GAL4 reduces triglyceride storage and induces Kennedy pathway enzymes and Xbp1 activation. (**A**) Third instar larval expression pattern of GFP driven by r4-GAL4 or cg-GAL4. (**B**) Transcript levels of *Drs*, normalized to *Rp49*, in fat bodies of late third instar larvae expressing GFP or Toll^10b^ under control of cg-GAL4, n = 8-9/group. ****p < 0.0001 versus GFP. (**C**) Whole-animal triglyceride levels, normalized to protein, in late third instar larvae expressing GFP or Toll^10b^ under control of cg-GAL4, n = 12/group. ****p < 0.0001 versus GFP. (**D**) *Lipin* (left) and *midway* (right) mRNA levels, normalized to *Rp49*, in late third instar fat bodies expressing GFP or Toll^10b^ under cg-GAL4 control, n = 8-9/group. ****p < 0.0001 versus GFP. (**E**) Transcript levels of *eas* (left) and *Pcyt1* (right), normalized to *Rp49*, in late third instar fat bodies expressing GFP or Toll^10b^ under cg-GAL4 control, n = 8-9/group. *p = 0.0220 and **p = 0.0025 versus GFP. (**F**) Western blot analysis of eas and Pcyt1 protein levels in fat bodies expressing GFP or Toll^10b^ under control of cg-GAL4. Histone H3 (HisH3) levels are shown as loading controls. (**G**) Spliced *Xbp1* (left) and *Hsc70-3* (*BiP*, right) mRNA levels, normalized to *Rp49*, in late third instar fat bodies expressing GFP or Toll^10b^ under cg-GAL4 control, n = 8-9/group. **p ≤ 0.0061 versus GFP. Data are presented as means ± SD. p values were determined by Student’s t test.
(TIF)

S4 Fig Toll signaling in larval fat body increases sensitivity to desiccation in adult flies. (**A**) Survival response to starvation with water and (**B**) survival response to starvation with desiccation in adult flies that expressed GFP or Toll^10b^ in fat body throughout the larval and pupal stages and as adults using r4-GAL4. Kaplan-Meier survival curves for females (left) and males (right) are shown for each stressor. For starvation with water, n = 30-33/group for females and n = 35-40/group for males. For starvation with desiccation, n = 18-27/group for females and n = 18-21/group for males. ****p < 0.0001 versus GFP. p values were determined by the log-rank test.
(TIF)

S5 Fig Lipin and midway are necessary for fat body triglyceride storage. For A-E, left panels: late third instar fat body levels of *Lipin* or *midway* mRNA, normalized to *Rp49*; right panels: late third instar whole-animal triglyceride levels, normalized to protein, in larvae of the indicated genotypes. (**A**) *Lipin* mRNA (n = 7/group) and triglycerides (n = 11-12/group) in larvae expressing GFP or Lipin^RNAi^ in fat body using r4-GAL4. ****p < 0.0001 versus GFP. (**B**) *midway* mRNA (n = 5-6/group) and triglycerides (n = 8/group) in larvae expressing GFP or mdy^RNAi^ in fat body. ****p < 0.0001 versus GFP. (**C**) *midway* mRNA (n = 6/group) and triglycerides (n = 8/group) in UAS-GFP/+; r4-GAL4/+ and *mdy*^*QX25*^/+; r4-GAL4/+ larvae. *p ≤ 0.0412 versus UAS-GFP/+; r4-GAL4/+. (**D**) *Lipin* mRNA (n = 5/group) and triglycerides (n = 8/group) in larvae expressing GFP or wild type Lipin in fat body. **p = 0.0089 versus GFP. (**E**) *midway* mRNA (n = 6-7/group) and triglycerides (n = 8/group) in larvae with r4-GAL4 driven expression of UAS-GFP or *mdy*^*EY07280*^ in fat body. ****p < 0.0001 versus GFP. (**F**) Left: Western blot of HA-tagged midway transgene expression in fat bodies expressing GFP or wild type, HA-tagged midway (UAS-mdy^HA^) under r4-GAL4 control (HA, top). Histone H3 (bottom) is shown as a loading control. Right: whole-animal triglycerides in larvae expressing GFP or mdy^HA^ in fat body, n = 12/group. (**G**) Triglyceride levels in CyO, GFP/+ and *mdy*^*QX25*^/+ larvae expressing GFP or mdy^HA^ in fat body, n = 6/group. **p = 0.0097 versus CyO, GFP/+; r4-GAL4/UAS-GFP. (**H**) *Lipin* (left) and *midway* (right) mRNA in larvae co-expressing wild type Lipin and HA-tagged midway with or without Toll^10b^ in fat body, n = 7/group. ****p < 0.0001 versus RFP+GFP. Data are presented as means ± SD. p values were determined by Student’s t test (A-F) and one-way ANOVA with Dunnett’s multiple comparison test (G, H).
(TIF)

S6 Fig Expression of Kennedy pathway enzymes and elevated levels of membrane phospholipids in fat bodies with active Toll signaling. (**A**) Late third instar fat body levels of *CG2201* transcripts, normalized to *Rp49*, n = 7/group. (**B**) Late third instar fat body levels of *CG33116* and *Pcyt2* transcripts, normalized to *Rp49*, n = 7/group. (**C**) Basal levels of Kennedy pathway enzymes in control fat bodies, measured by RNA-Sequencing. Normalized read count data shown are from Suzawa et al., 2019. (**D**) Representative image of TLC separation of PE (top) and PC (bottom) from pooled larval fat bodies (n = 8/sample) expressing GFP or Toll^10b^ driven by r4-GAL4. Standards are outlined in red. (**E**) Area of PE and PC from densitometry of iodine-stained TLC plates, normalized to total protein in lysates, n = 8 measurements/genotype from three separate experiments. ***p = 0.0007 versus GFP (PE) and *p = 0.0146 versus GFP (PC). Data are presented as means ± SD. p values were determined by Student’s t test.
(TIF)

S7 Fig Dif is sufficient to regulate expression of lipid metabolic enzymes and *Xbp1* splicing in fat body. (**A**) *eas* (left) and *Pcyt1* (right) mRNA levels in late third instar larval fat bodies expressing GFP or Dif, n = 4-6/group. **p = 0.0011 and ***p = 0.0003 versus GFP. (**B**) *Lipin* (left) and *midway* (right) mRNA levels in late third instar larval fat bodies expressing GFP or Dif, n = 4-6/group. *p = 0.0470 and **p = 0.0047 versus GFP. (**C**) Transcript levels of spliced *Xbp1* in late third instar larval fat bodies with r4-GAL4-driven expression of (left) GFP or Dif, n = 4-6/group, **p = 0.0049 versus GFP; or (right) GFP or Toll^10b^+Dif^RNAi^, n = 4-5/group. All transcripts were normalized to *Rp49*. Data are presented as means ± SD. p values were determined by Student’s unpaired t test.
(TIF)

S8 Fig Toll signaling induces Kennedy pathway enzymes independently of SREBP. (**A**) Late third instar fat body *SREBP* transcript levels, normalized to *Rp49*, in fat bodies expressing RFP or Toll^10b^ with GFP or SREBP^RNAi^ under control of r4-GAL4, n = 5-6/group. **p ≤ 0.0069, ***p = 0.0003, and ****p < 0.0001 versus RFP+GFP. (**B**) Western blot of SREBP (top) and Histone H3 (bottom) in fat bodies of the indicated genotypes. (**C**) Late third instar fat body mRNA levels of Kennedy pathway enzymes *eas*, *CG7149*, *CG2201* and *Pcyt1*, normalized to *Rp49*, in fat bodies of the indicated genotypes, n = 5-6/group. *p = 0.0139, **p ≤ 0.0073, ***p ≤ 0.0006, and ****p < 0.0001 versus RFP+GFP. (**D**) Late third instar fat body mRNA levels of unspliced and spliced *Xbp1* and *Drs*, normalized to *Rp49*, in fat bodies of the indicated genotypes, n = 5-6/group. *p ≤ 0.0155, **p ≤ 0.0096, and ****p < 0.0001 versus RFP+GFP. (**E**) Transcript levels of *SREBP* and *Drs* were measured by RT-qPCR in late third instar larval fat bodies with GAL80ts-mediated induction of Toll^10b^ with or without Xbp1^RNAi^ for 24 hours at 30°C, n = 6/group. *p ≤ 0.0366 and ****p < 0.0001 versus fat bodies acutely expressing RFP+GFP. Data are presented as means ± SD. p values were determined by one-way ANOVA with the Tukey-Kramer multiple comparisons test (A, C-E).
(TIF)

S9 Fig Atf6, but not PEK, plays a minor role in Kennedy pathway gene regulation in response to Toll signaling. (**A**) Transcript levels of indicated genes in late third instar larval fat bodies expressing Toll^10b^ with or without PEK^RNAi^ under control of r4-GAL4, n = 6-7/group. ****p < 0.0001 versus RFP+GFP controls and ****p < 0.0001 for Toll^10b^+GFP versus Toll^10b^+PEK^RNAi^. (**B**) Transcript levels of indicated genes in late third instar larval fat bodies expressing Toll^10b^ with or without Atf6^RNAi^, n = 7/group. *p ≤ 0.0321, **p ≤ 0.0059, ***p ≤ 0.0007, ****p < 0.0001 versus RFP+GFP controls and ****p < 0.0001 for Toll^10b^+GFP versus Toll^10b^+Atf6^RNAi^. All transcripts were measured by RT-qPCR and normalized to *Rp49*. Data are presented as means ± SD. p values were determined by one-way ANOVA with the Tukey-Kramer multiple comparisons test.
(TIF)

S10 Fig Untargeted immune molecules are induced in fat bodies with *Bom*^*Δ55C*^ and *Drs*^*Δ7–17*^ mutations. (**A**) Expression levels of AMPs in control fat bodies expressing RFP and in immune-activated fat bodies expressing Toll^10b^ under r4-GAL4 control. Normalized read count data from RNA-sequencing published in Suzawa et al., 2019 are shown. AMPs clustered on chromosome 2 (deleted in the *Bom*^*Δ55C*^ mutant, orange line) represent 70% of the AMP transcripts induced by Toll^10b^ expression. Drs, deleted in the *Drs*^*Δ7–17*^ mutant, represents 10% of the induced AMP transcripts. (**B**) Transcript levels of *Daisho1* (*IM4*) (top) and *Daisho2* (*IM14*) (bottom), normalized to *Rp49*, in fat bodies of late third instar larvae expressing GFP (closed symbols) or Toll^10b^ (open symbols) under r4-GAL4 control. Animals were wild type, heterozygous, or homozygous for *Drs*^*Δ7–17*^ and *Bom*^*Δ55C*^ as indicated, n = 5-8/group. **p = 0.0015 and ****p < 0.0001 versus GFP-expressing controls with the same *Drs*^*Δ7–17*^ and *Bom*^*Δ55C*^ genotypes. Note that GFP-expressing controls are heterozygous for *Drs*^*Δ7–17*^ while Toll^10b^-expressing larvae are homozygous for *Drs*^*Δ7–17*^. Data are presented as means ± SD. p values were determined by Student’s t test.
(TIF)

S11 Fig Knockdown of Pcyt1 and eas in fat body induces *Xbp1* splicing and expression of Xbp1 target genes. (**A**) Western blot of Pcyt1 and eas in lysates from late third instar larval fat bodies expressing GFP, Toll^10b^, RFP+GFP controls or Pcyt1^RNAi^+eas^RNAi^ transgenes with or without Toll^10b^ under control of r4-GAL4. Histone H3 levels are shown as loading controls. (**B**) Triglyceride levels in late third instar larvae expressing RFP+GFP or Pcyt1^RNAi^ +eas^RNAi^ with or without Toll^10b^ in fat body under control of r4-GAL4, n = 8/group. ***p = 0.0003 versus RFP+GFP control. (**C**) Transcript levels of spliced *Xbp1* and Xbp1 targets *Hsc70-3 (BiP)*, *Pdi*, and *Sec24cd* in late third instar fat bodies expressing RFP+GFP or Pcyt1^RNAi^ +eas^RNAi^ with or without Toll^10b^ using r4-GAL4, n = 5-7/group. *p ≤ 0.0265, **p = 0.0012, ***p = 0.00023, and ****p < 0.0001 versus RFP+GFP. Data are presented as means ± SD. p values were determined by Student’s unpaired t test (B, C).
(TIF)

S1 Table Genotypes of *Drosophila melanogaster* used in this study. (DOCX)

S2 Table Sequences of oligonucleotides used in this study. (DOCX)
