# Supplementary figures and images for: Innate immune signaling in Drosophila shifts anabolic lipid metabolism from triglyceride storage to phospholipid synthesis to support immune function
Source: PLoS Genet. 2020 Nov 23;16(11):e1009192. doi: 10.1371/journal.pgen.1009192 (PMC7721134; doi:10.1371/journal.pgen.1009192)

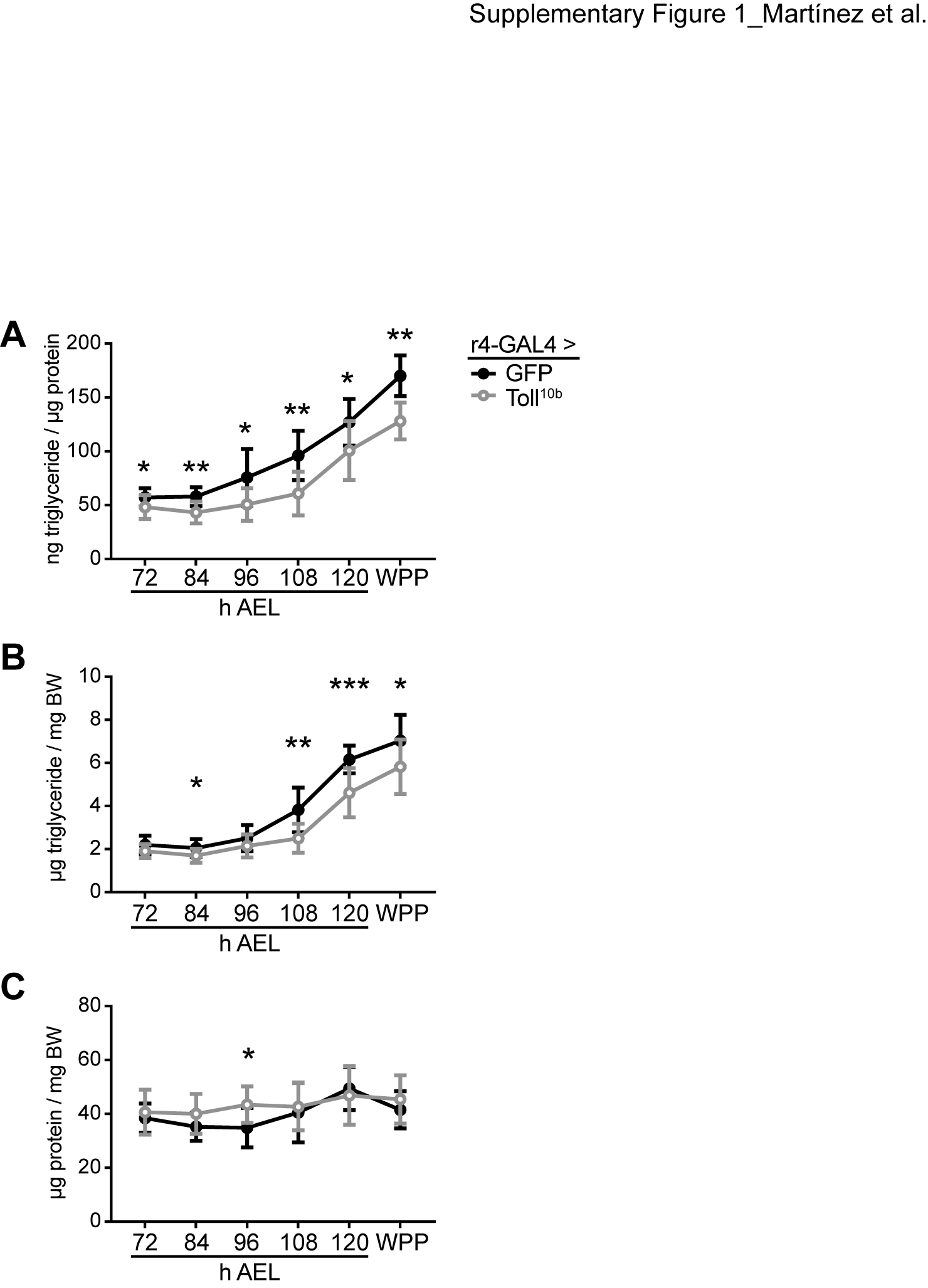

Supplement: S1 Fig — (A) Whole-animal triglyceride levels throughout the third instar (72–120 hours after egg lay (h AEL)) and in white prepupae (WPP) were normalized to protein, n = 10-11/group. *p ≤ 0.0459 and **p ≤ 0.0019 versus GFP. (B) Whole-animal triglyceride levels throughout the third instar and in white prepupae were normalized to body weight, n = 10-11/group. *p ≤ 0.0373, **p = 0.0033, and ***p = 0.0009 versus GFP. (C) Whole-animal protein levels throughout the third instar and in white prepupae were normalized to body weight, n = 10-11/group. *p = 0.0117 versus GFP. Data are presented as means ± SD. p values were determined by Student’s unpaired t test. (TIF) [file pgen.1009192.s001.tif]

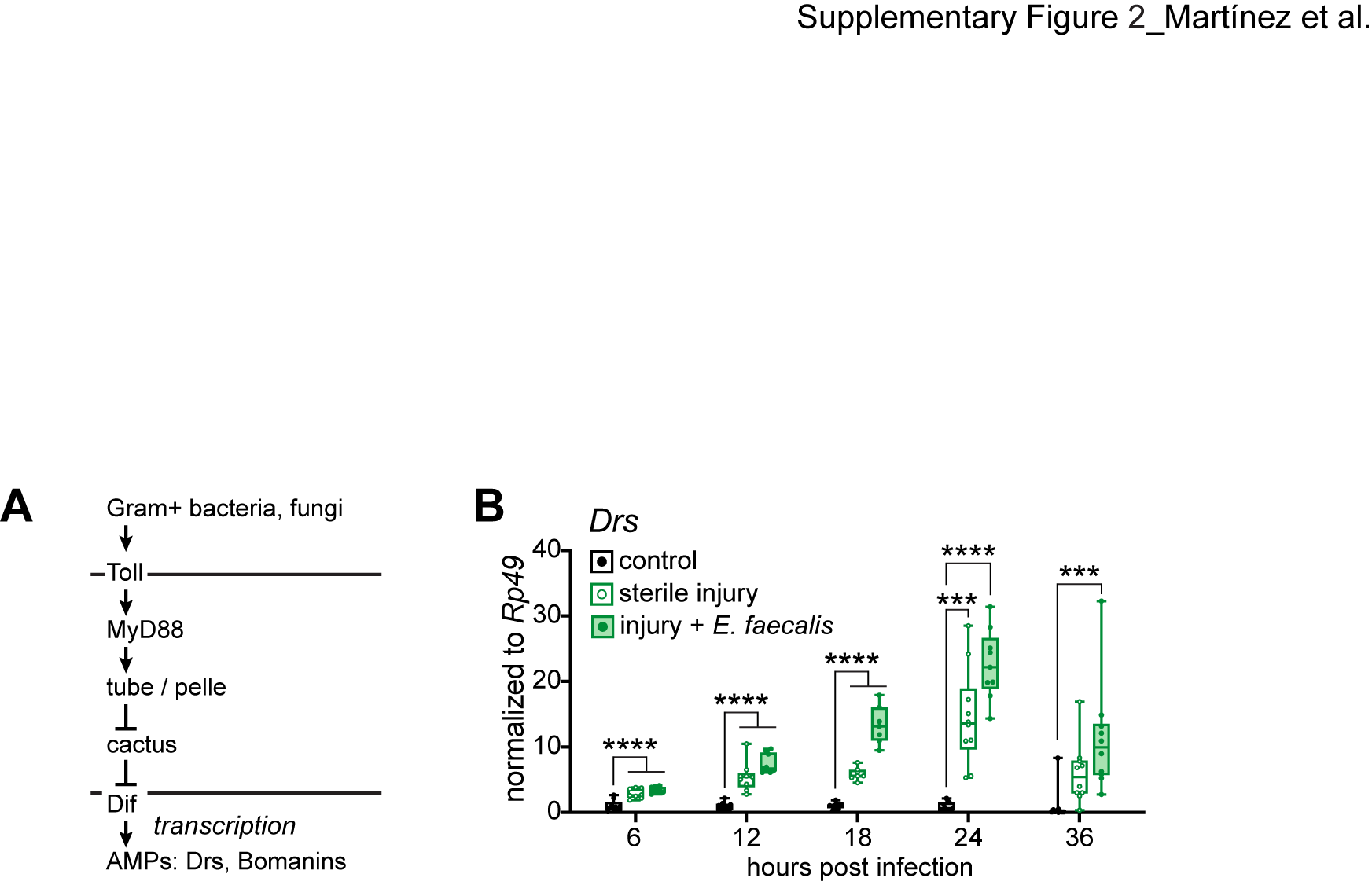

Supplement: S2 Fig — (A) Schematic representation of the Toll signaling pathway leading to AMP expression. In response to activation of Toll receptors, the IκB homolog cactus is phosphorylated and degraded, freeing Dif to translocate into the nucleus to regulate expression of canonical targets such as the antimicrobial peptide genes encoding Drosomycin and the Bomanin peptides. (B) Transcript levels of Drs, normalized to Rp49, in third instar larval fat bodies from uninfected controls, larvae subjected to sterile injury, and larvae infected with Enterococcus faecalis at 6–36 hours post infection n = 7-10/group. ***p ≤ 0.0009 and ****p < 0.0001 versus uninfected controls. Transcripts were normalized to Rp49. Data are presented as means ± SD. p values were determined by one-way ANOVA with Dunnett’s multiple comparisons test. (TIF) [file pgen.1009192.s002.tif]

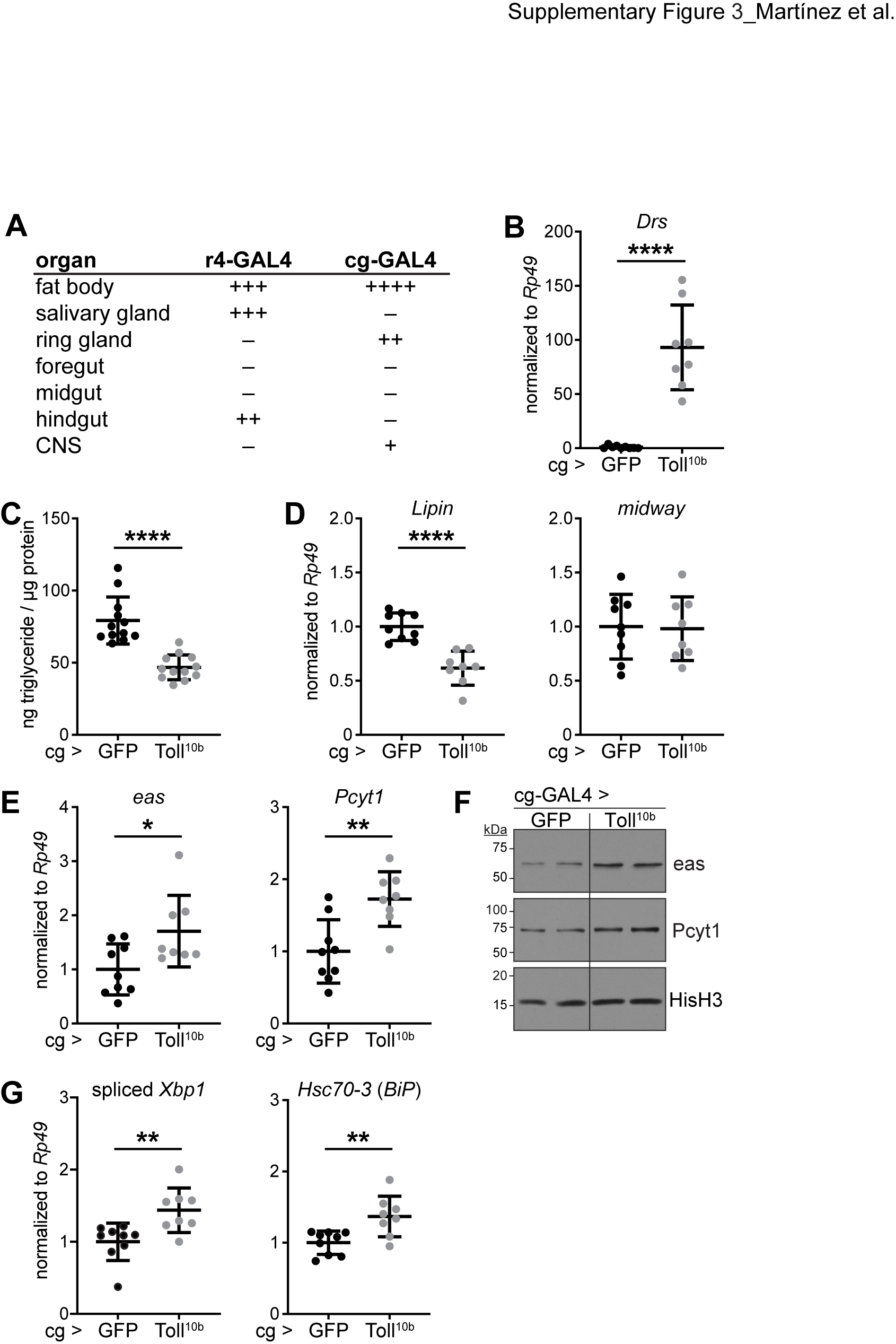

Supplement: S3 Fig — (A) Third instar larval expression pattern of GFP driven by r4-GAL4 or cg-GAL4. (B) Transcript levels of Drs, normalized to Rp49, in fat bodies of late third instar larvae expressing GFP or Toll10b under control of cg-GAL4, n = 8-9/group. ****p < 0.0001 versus GFP. (C) Whole-animal triglyceride levels, normalized to protein, in late third instar larvae expressing GFP or Toll10b under control of cg-GAL4, n = 12/group. ****p < 0.0001 versus GFP. (D) Lipin (left) and midway (right) mRNA levels, normalized to Rp49, in late third instar fat bodies expressing GFP or Toll10b under cg-GAL4 control, n = 8-9/group. ****p < 0.0001 versus GFP. (E) Transcript levels of eas (left) and Pcyt1 (right), normalized to Rp49, in late third instar fat bodies expressing GFP or Toll10b under cg-GAL4 control, n = 8-9/group. *p = 0.0220 and **p = 0.0025 versus GFP. (F) Western blot analysis of eas and Pcyt1 protein levels in fat bodies expressing GFP or Toll10b under control of cg-GAL4. Histone H3 (HisH3) levels are shown as loading controls. (G) Spliced Xbp1 (left) and Hsc70-3 (BiP, right) mRNA levels, normalized to Rp49, in late third instar fat bodies expressing GFP or Toll10b under cg-GAL4 control, n = 8-9/group. **p ≤ 0.0061 versus GFP. Data are presented as means ± SD. p values were determined by Student’s t test. (TIF) [file pgen.1009192.s003.tif]

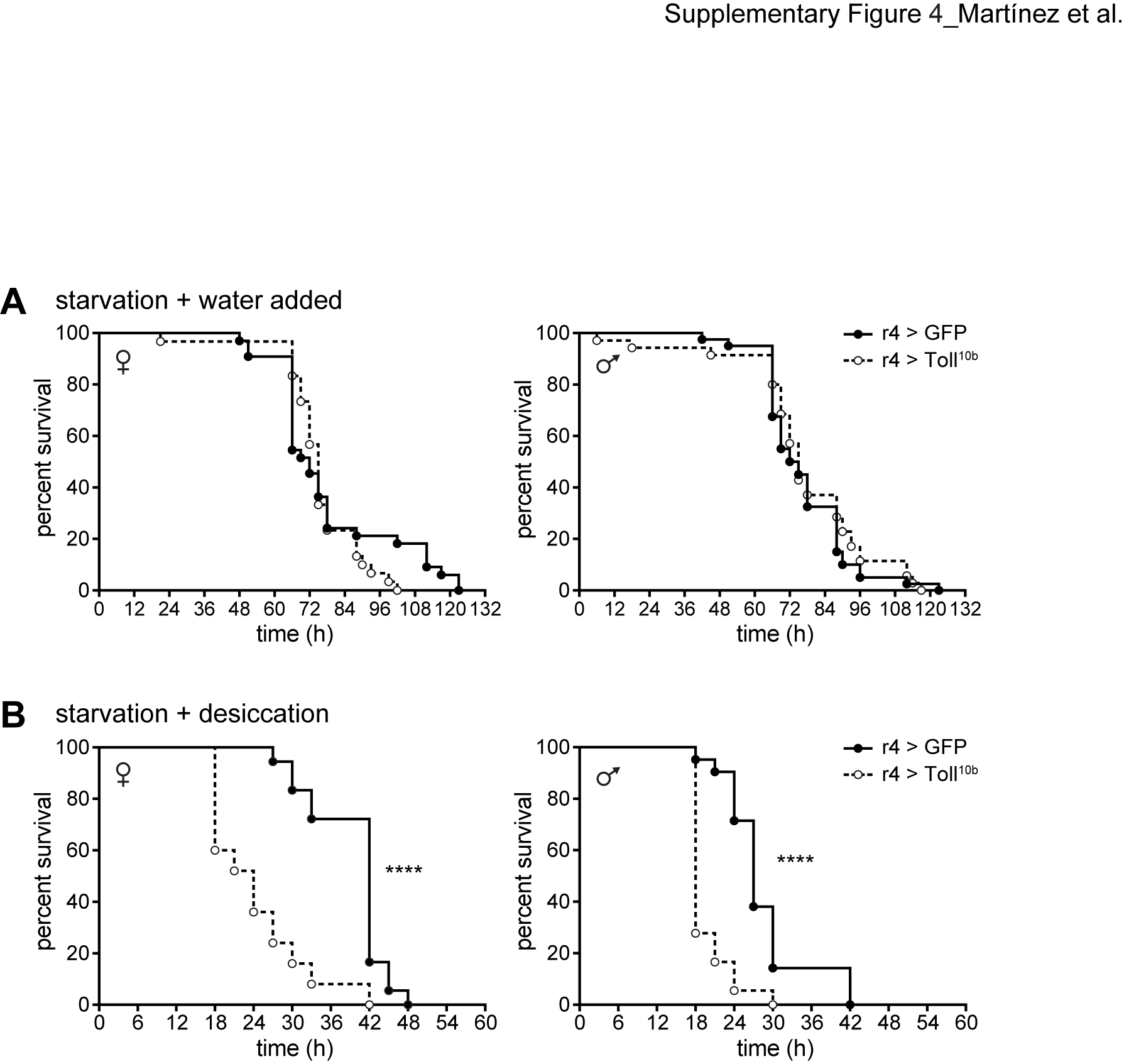

Supplement: S4 Fig — (A) Survival response to starvation with water and (B) survival response to starvation with desiccation in adult flies that expressed GFP or Toll10b in fat body throughout the larval and pupal stages and as adults using r4-GAL4. Kaplan-Meier survival curves for females (left) and males (right) are shown for each stressor. For starvation with water, n = 30-33/group for females and n = 35-40/group for males. For starvation with desiccation, n = 18-27/group for females and n = 18-21/group for males. ****p < 0.0001 versus GFP. p values were determined by the log-rank test. (TIF) [file pgen.1009192.s004.tif]

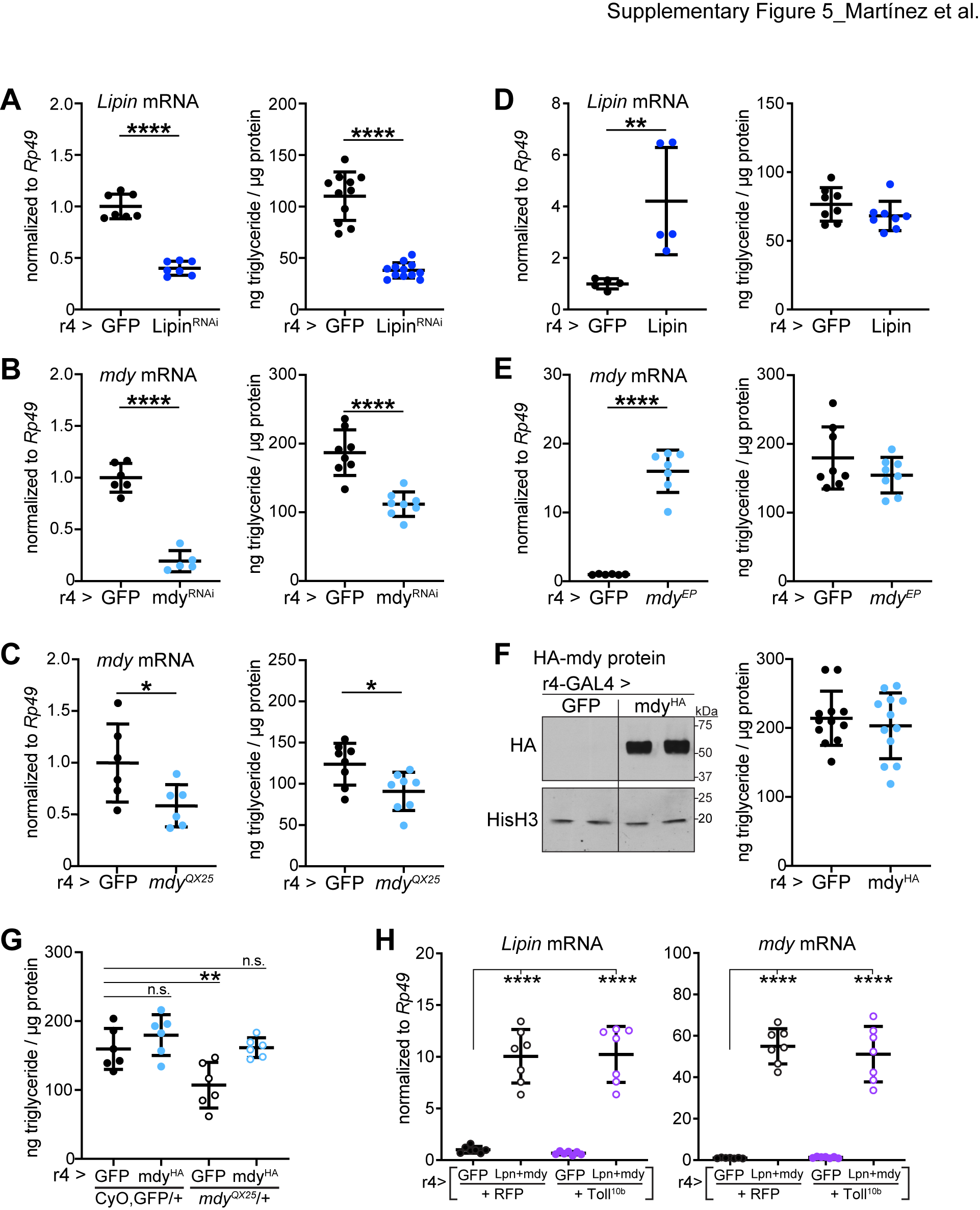

Supplement: S5 Fig — For A-E, left panels: late third instar fat body levels of Lipin or midway mRNA, normalized to Rp49; right panels: late third instar whole-animal triglyceride levels, normalized to protein, in larvae of the indicated genotypes. (A) Lipin mRNA (n = 7/group) and triglycerides (n = 11-12/group) in larvae expressing GFP or LipinRNAi in fat body using r4-GAL4. ****p < 0.0001 versus GFP. (B) midway mRNA (n = 5-6/group) and triglycerides (n = 8/group) in larvae expressing GFP or mdyRNAi in fat body. ****p < 0.0001 versus GFP. (C) midway mRNA (n = 6/group) and triglycerides (n = 8/group) in UAS-GFP/+; r4-GAL4/+ and mdyQX25/+; r4-GAL4/+ larvae. *p ≤ 0.0412 versus UAS-GFP/+; r4-GAL4/+. (D) Lipin mRNA (n = 5/group) and triglycerides (n = 8/group) in larvae expressing GFP or wild type Lipin in fat body. **p = 0.0089 versus GFP. (E) midway mRNA (n = 6-7/group) and triglycerides (n = 8/group) in larvae with r4-GAL4 driven expression of UAS-GFP or mdyEY07280 in fat body. ****p < 0.0001 versus GFP. (F) Left: Western blot of HA-tagged midway transgene expression in fat bodies expressing GFP or wild type, HA-tagged midway (UAS-mdyHA) under r4-GAL4 control (HA, top). Histone H3 (bottom) is shown as a loading control. Right: whole-animal triglycerides in larvae expressing GFP or mdyHA in fat body, n = 12/group. (G) Triglyceride levels in CyO, GFP/+ and mdyQX25/+ larvae expressing GFP or mdyHA in fat body, n = 6/group. **p = 0.0097 versus CyO, GFP/+; r4-GAL4/UAS-GFP. (H) Lipin (left) and midway (right) mRNA in larvae co-expressing wild type Lipin and HA-tagged midway with or without Toll10b in fat body, n = 7/group. ****p < 0.0001 versus RFP+GFP. Data are presented as means ± SD. p values were determined by Student’s t test (A-F) and one-way ANOVA with Dunnett’s multiple comparison test (G, H). (TIF) [file pgen.1009192.s005.tif]

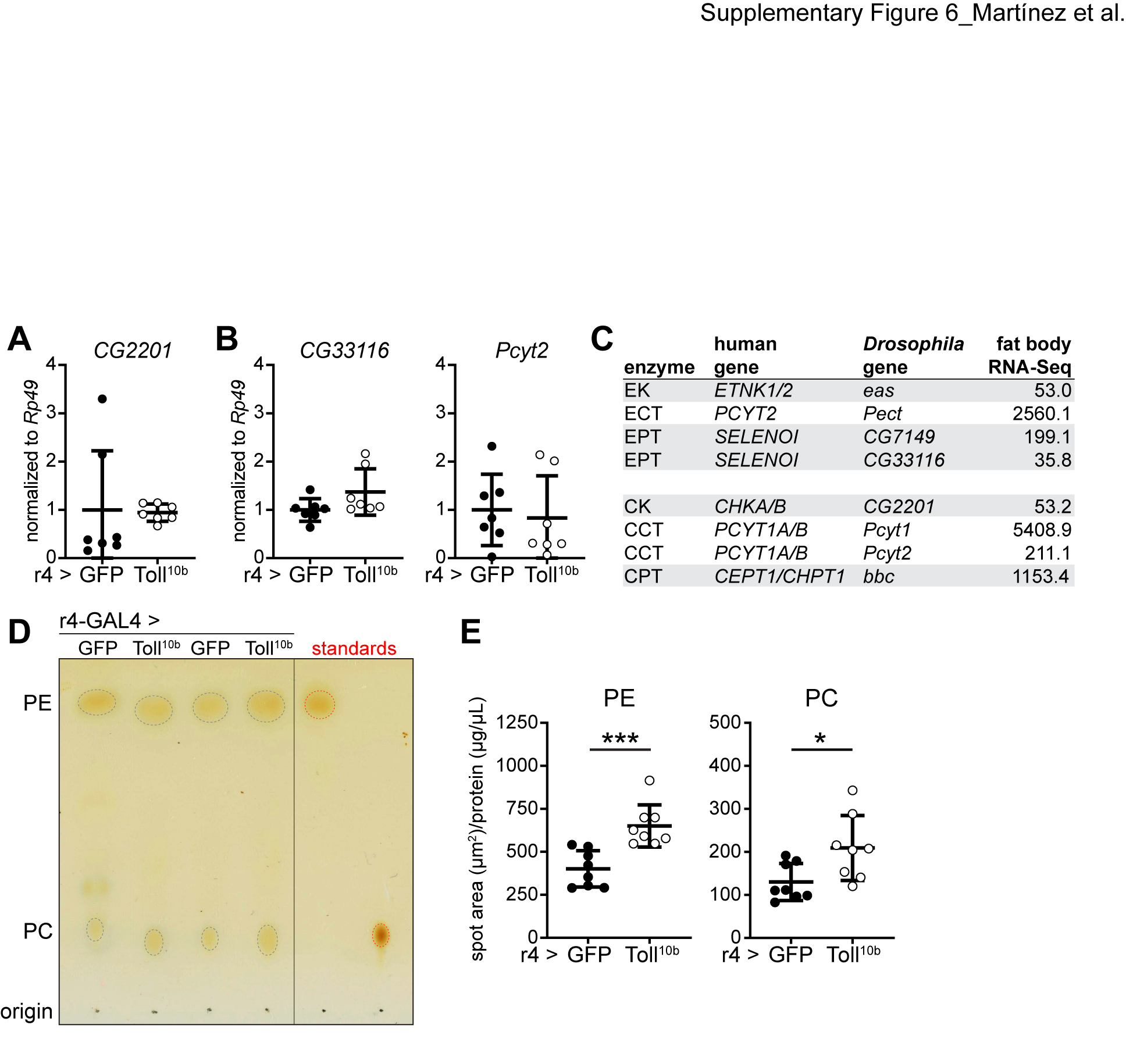

Supplement: S6 Fig — (A) Late third instar fat body levels of CG2201 transcripts, normalized to Rp49, n = 7/group. (B) Late third instar fat body levels of CG33116 and Pcyt2 transcripts, normalized to Rp49, n = 7/group. (C) Basal levels of Kennedy pathway enzymes in control fat bodies, measured by RNA-Sequencing. Normalized read count data shown are from Suzawa et al., 2019. (D) Representative image of TLC separation of PE (top) and PC (bottom) from pooled larval fat bodies (n = 8/sample) expressing GFP or Toll10b driven by r4-GAL4. Standards are outlined in red. (E) Area of PE and PC from densitometry of iodine-stained TLC plates, normalized to total protein in lysates, n = 8 measurements/genotype from three separate experiments. ***p = 0.0007 versus GFP (PE) and *p = 0.0146 versus GFP (PC). Data are presented as means ± SD. p values were determined by Student’s t test. (TIF) [file pgen.1009192.s006.tif]

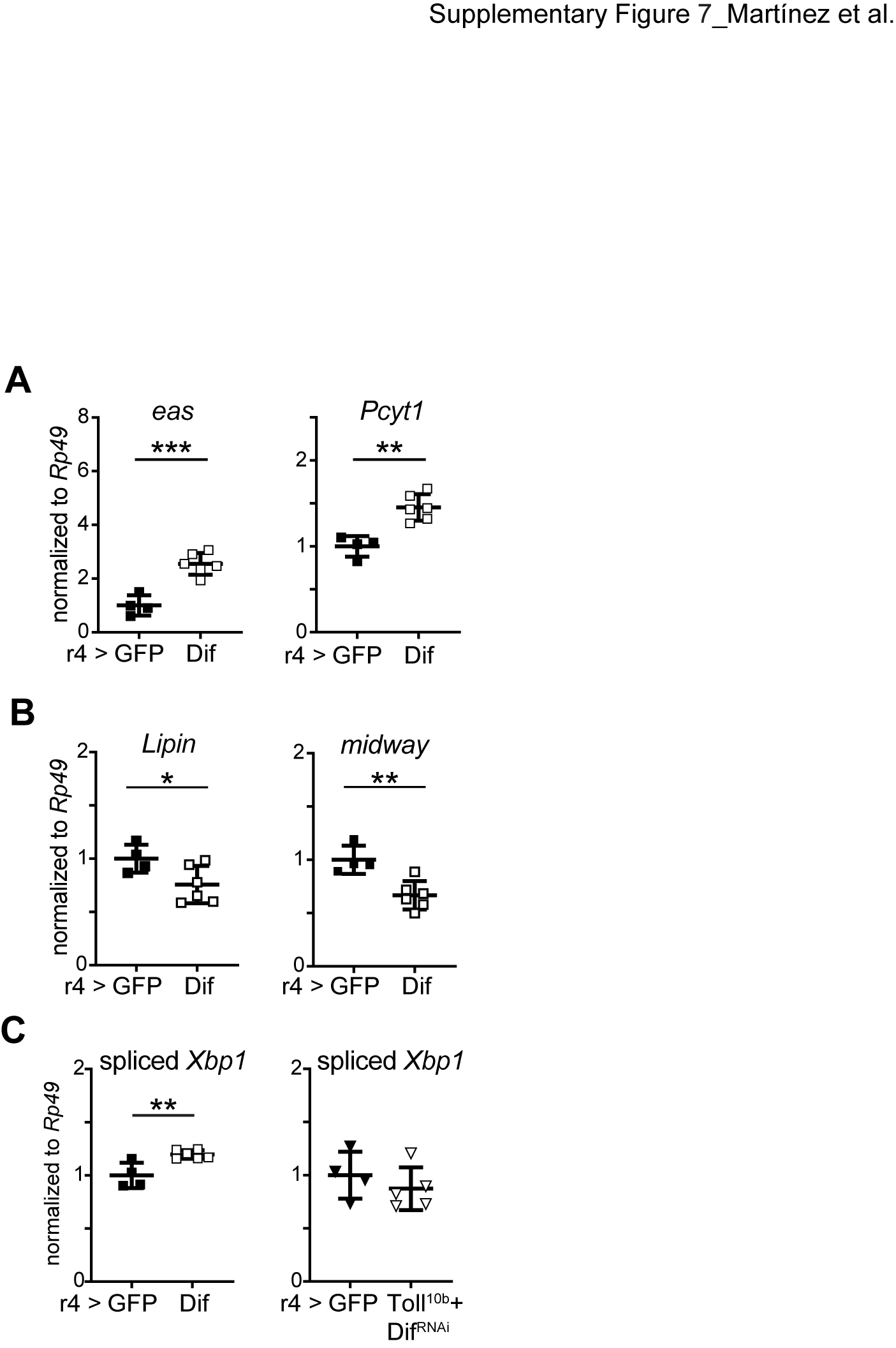

Supplement: S7 Fig — (A) eas (left) and Pcyt1 (right) mRNA levels in late third instar larval fat bodies expressing GFP or Dif, n = 4-6/group. **p = 0.0011 and ***p = 0.0003 versus GFP. (B) Lipin (left) and midway (right) mRNA levels in late third instar larval fat bodies expressing GFP or Dif, n = 4-6/group. *p = 0.0470 and **p = 0.0047 versus GFP. (C) Transcript levels of spliced Xbp1 in late third instar larval fat bodies with r4-GAL4-driven expression of (left) GFP or Dif, n = 4-6/group, **p = 0.0049 versus GFP; or (right) GFP or Toll10b+DifRNAi, n = 4-5/group. All transcripts were normalized to Rp49. Data are presented as means ± SD. p values were determined by Student’s unpaired t test. (TIF) [file pgen.1009192.s007.tif]

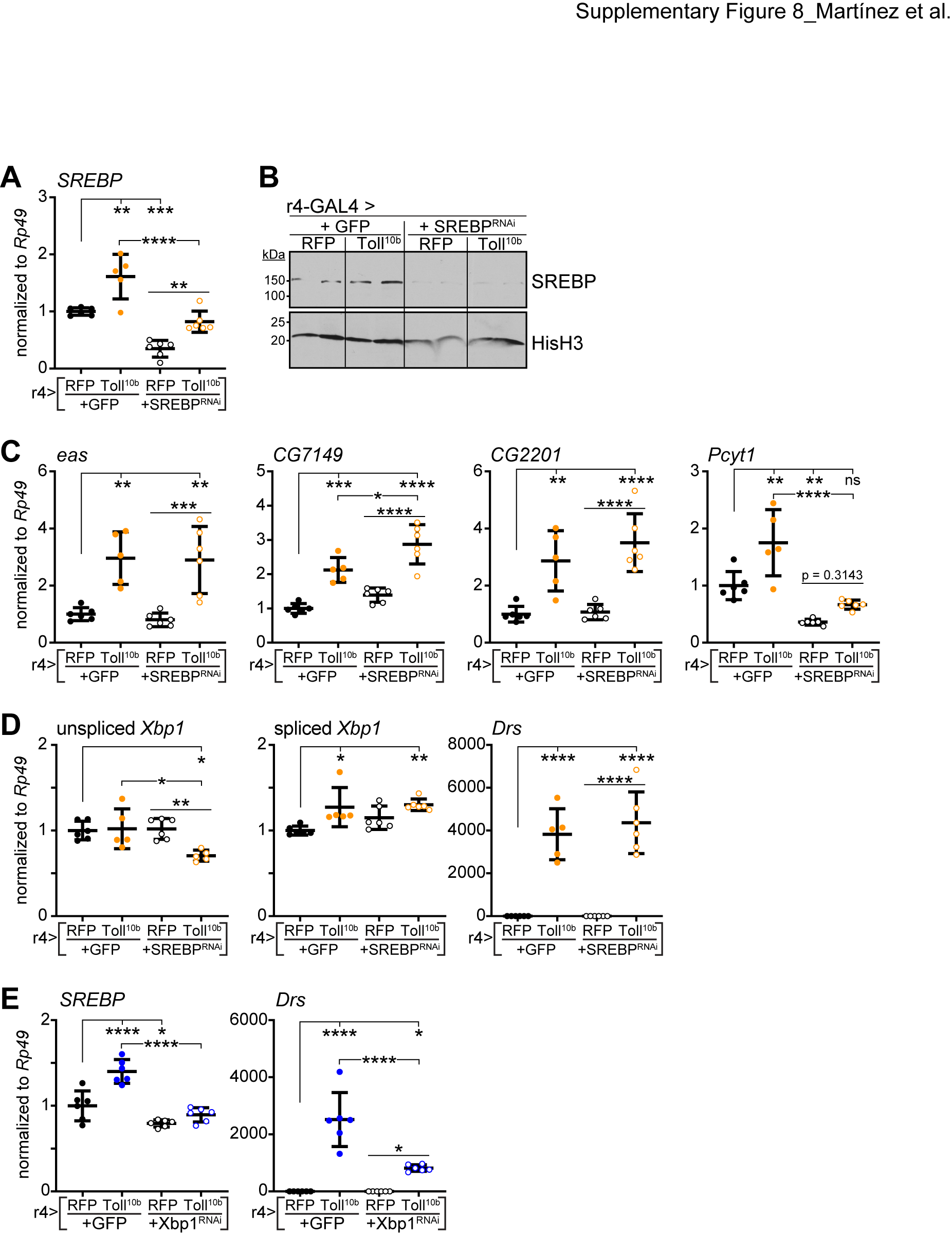

Supplement: S8 Fig — (A) Late third instar fat body SREBP transcript levels, normalized to Rp49, in fat bodies expressing RFP or Toll10b with GFP or SREBPRNAi under control of r4-GAL4, n = 5-6/group. **p ≤ 0.0069, ***p = 0.0003, and ****p < 0.0001 versus RFP+GFP. (B) Western blot of SREBP (top) and Histone H3 (bottom) in fat bodies of the indicated genotypes. (C) Late third instar fat body mRNA levels of Kennedy pathway enzymes eas, CG7149, CG2201 and Pcyt1, normalized to Rp49, in fat bodies of the indicated genotypes, n = 5-6/group. *p = 0.0139, **p ≤ 0.0073, ***p ≤ 0.0006, and ****p < 0.0001 versus RFP+GFP. (D) Late third instar fat body mRNA levels of unspliced and spliced Xbp1 and Drs, normalized to Rp49, in fat bodies of the indicated genotypes, n = 5-6/group. *p ≤ 0.0155, **p ≤ 0.0096, and ****p < 0.0001 versus RFP+GFP. (E) Transcript levels of SREBP and Drs were measured by RT-qPCR in late third instar larval fat bodies with GAL80ts-mediated induction of Toll10b with or without Xbp1RNAi for 24 hours at 30°C, n = 6/group. *p ≤ 0.0366 and ****p < 0.0001 versus fat bodies acutely expressing RFP+GFP. Data are presented as means ± SD. p values were determined by one-way ANOVA with the Tukey-Kramer multiple comparisons test (A, C-E). (TIF) [file pgen.1009192.s008.tif]

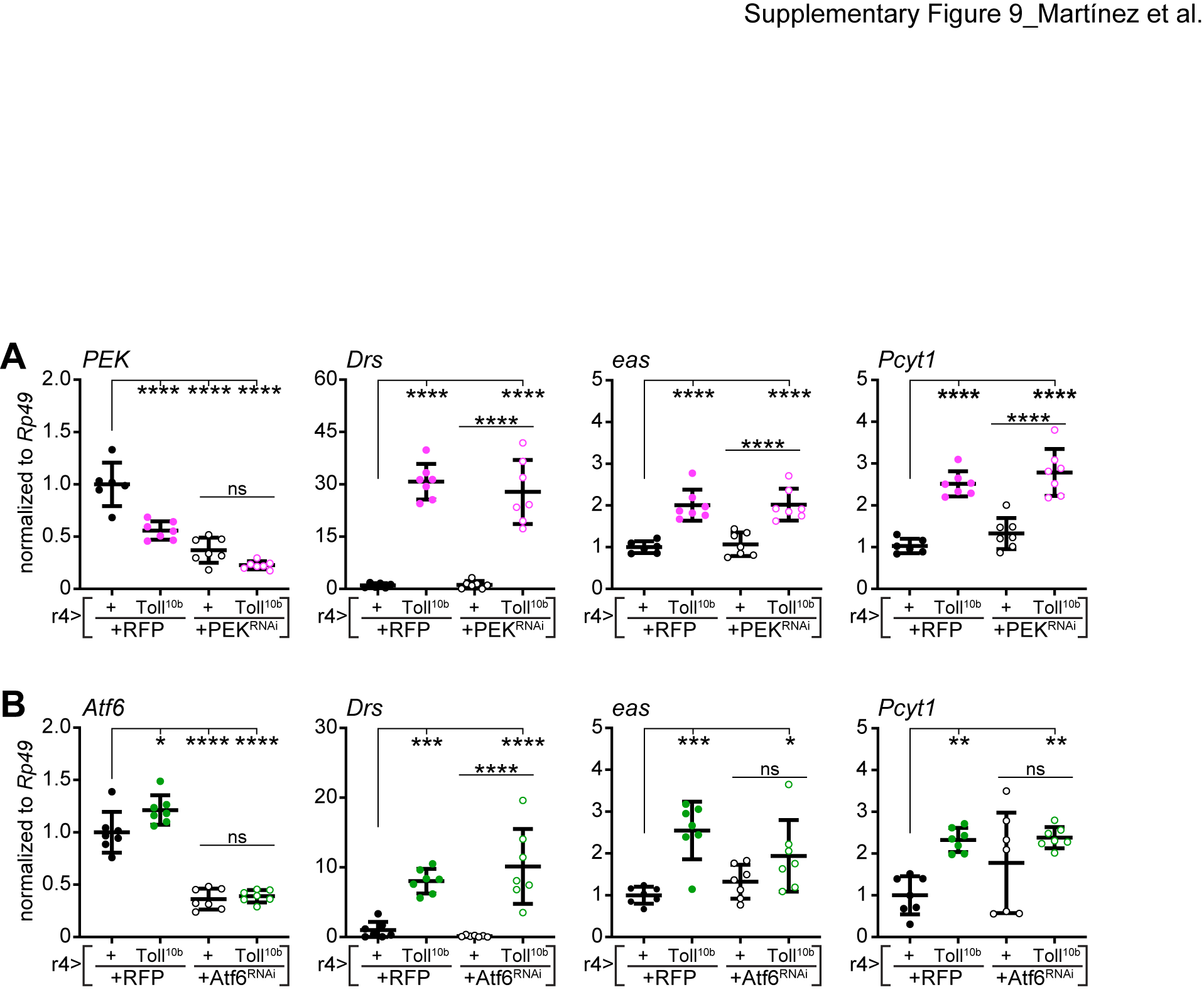

Supplement: S9 Fig — (A) Transcript levels of indicated genes in late third instar larval fat bodies expressing Toll10b with or without PEKRNAi under control of r4-GAL4, n = 6-7/group. ****p < 0.0001 versus RFP+GFP controls and ****p < 0.0001 for Toll10b+GFP versus Toll10b+PEKRNAi. (B) Transcript levels of indicated genes in late third instar larval fat bodies expressing Toll10b with or without Atf6RNAi, n = 7/group. *p ≤ 0.0321, **p ≤ 0.0059, ***p ≤ 0.0007, ****p < 0.0001 versus RFP+GFP controls and ****p < 0.0001 for Toll10b+GFP versus Toll10b+Atf6RNAi. All transcripts were measured by RT-qPCR and normalized to Rp49. Data are presented as means ± SD. p values were determined by one-way ANOVA with the Tukey-Kramer multiple comparisons test. (TIF) [file pgen.1009192.s009.tif]

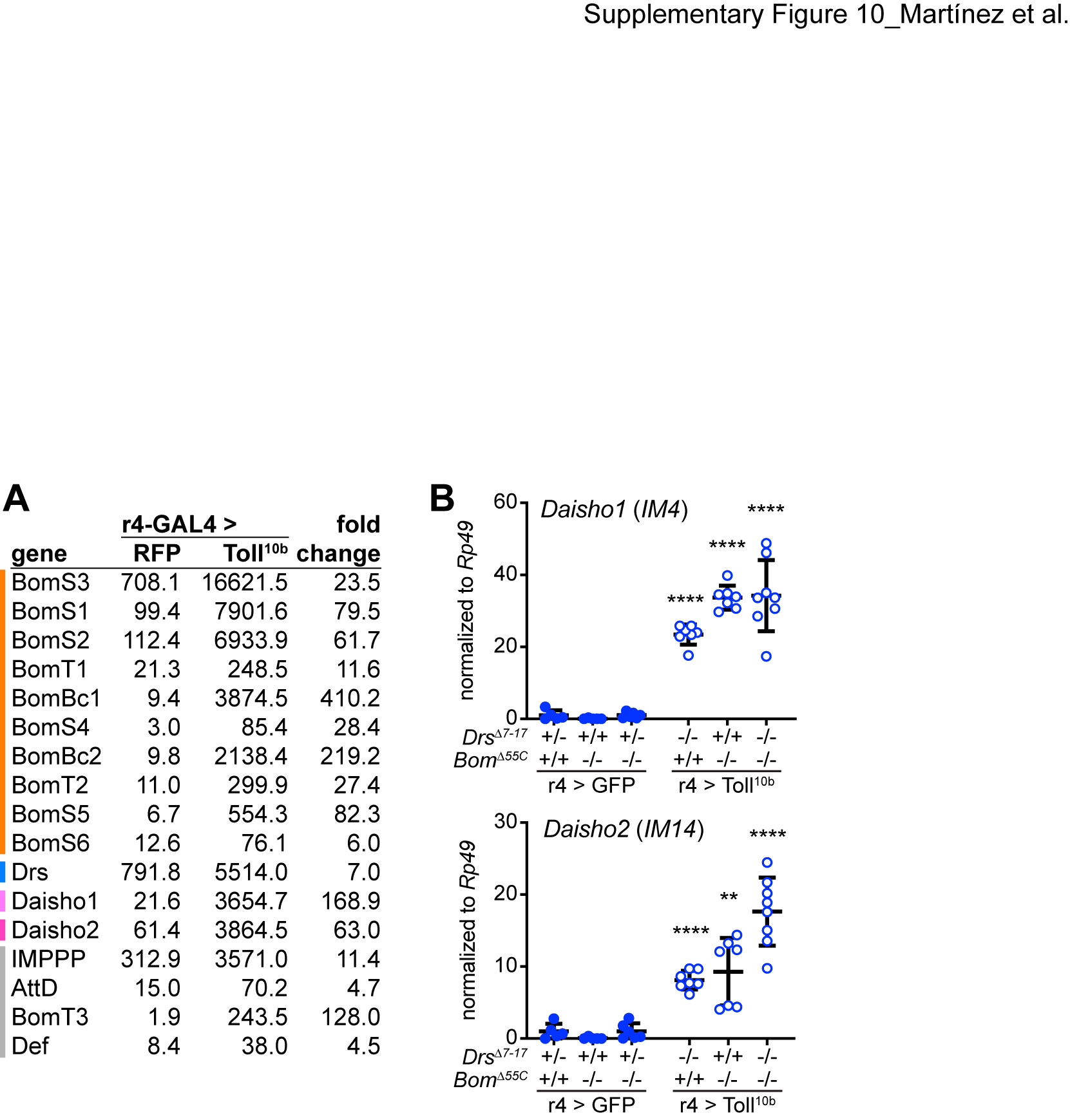

Supplement: S10 Fig — (A) Expression levels of AMPs in control fat bodies expressing RFP and in immune-activated fat bodies expressing Toll10b under r4-GAL4 control. Normalized read count data from RNA-sequencing published in Suzawa et al., 2019 are shown. AMPs clustered on chromosome 2 (deleted in the BomΔ55C mutant, orange line) represent 70% of the AMP transcripts induced by Toll10b expression. Drs, deleted in the DrsΔ7–17 mutant, represents 10% of the induced AMP transcripts. (B) Transcript levels of Daisho1 (IM4) (top) and Daisho2 (IM14) (bottom), normalized to Rp49, in fat bodies of late third instar larvae expressing GFP (closed symbols) or Toll10b (open symbols) under r4-GAL4 control. Animals were wild type, heterozygous, or homozygous for DrsΔ7–17 and BomΔ55C as indicated, n = 5-8/group. **p = 0.0015 and ****p < 0.0001 versus GFP-expressing controls with the same DrsΔ7–17 and BomΔ55C genotypes. Note that GFP-expressing controls are heterozygous for DrsΔ7–17 while Toll10b-expressing larvae are homozygous for DrsΔ7–17. Data are presented as means ± SD. p values were determined by Student’s t test. (TIF) [file pgen.1009192.s010.tif]

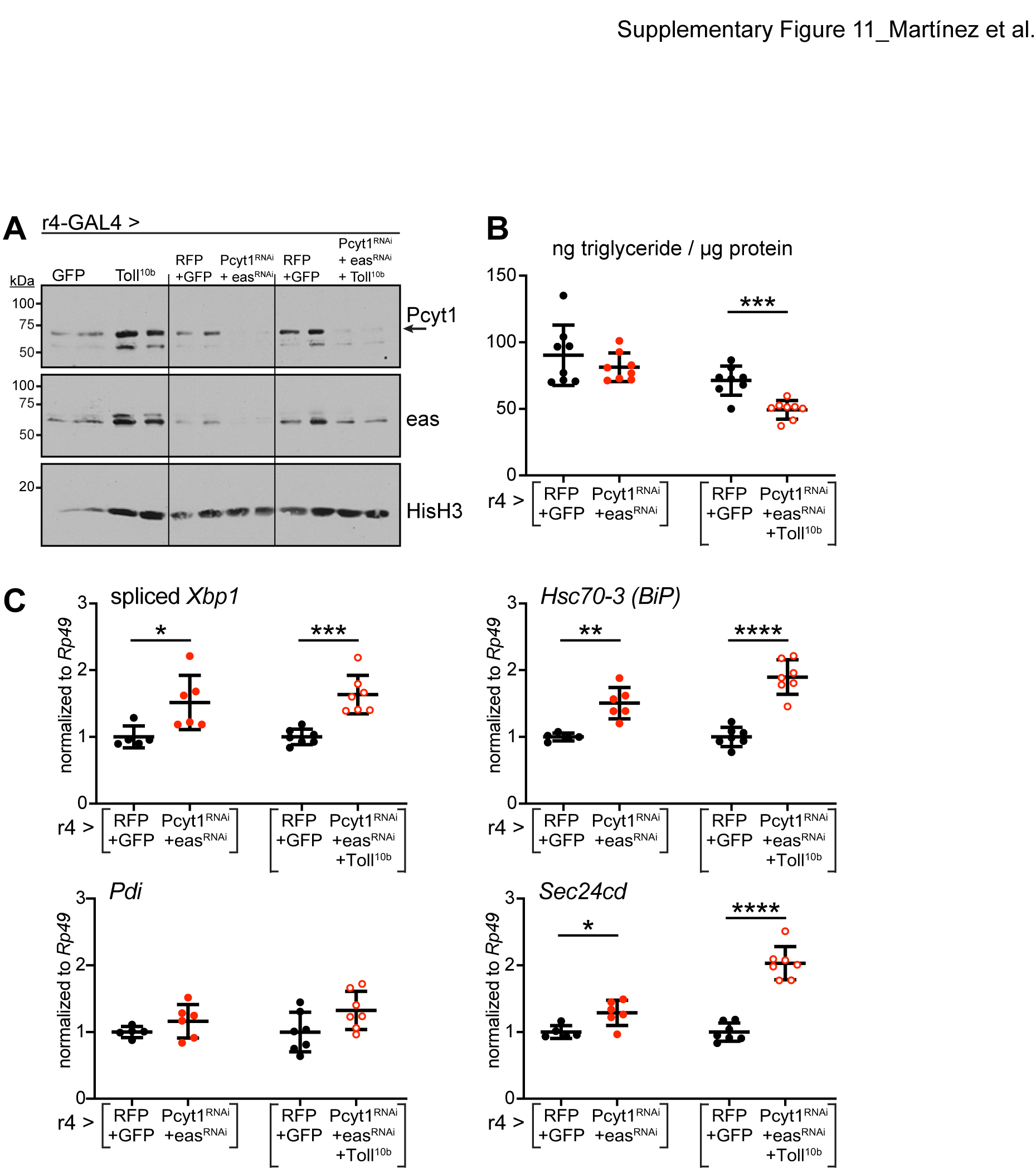

Supplement: S11 Fig — (A) Western blot of Pcyt1 and eas in lysates from late third instar larval fat bodies expressing GFP, Toll10b, RFP+GFP controls or Pcyt1RNAi+easRNAi transgenes with or without Toll10b under control of r4-GAL4. Histone H3 levels are shown as loading controls. (B) Triglyceride levels in late third instar larvae expressing RFP+GFP or Pcyt1RNAi +easRNAi with or without Toll10b in fat body under control of r4-GAL4, n = 8/group. ***p = 0.0003 versus RFP+GFP control. (C) Transcript levels of spliced Xbp1 and Xbp1 targets Hsc70-3 (BiP), Pdi, and Sec24cd in late third instar fat bodies expressing RFP+GFP or Pcyt1RNAi +easRNAi with or without Toll10b using r4-GAL4, n = 5-7/group. *p ≤ 0.0265, **p = 0.0012, ***p = 0.00023, and ****p < 0.0001 versus RFP+GFP. Data are presented as means ± SD. p values were determined by Student’s unpaired t test (B, C). (TIF) [file pgen.1009192.s011.tif]
