# Supplementary material for: Innate immune signaling in Drosophila shifts anabolic lipid metabolism from triglyceride storage to phospholipid synthesis to support immune function
Source: PLoS Genet. 2020 Nov 23;16(11):e1009192. doi: 10.1371/journal.pgen.1009192 (PMC7721134; doi:10.1371/journal.pgen.1009192)
Supplement: S1 Table — (DOCX) [file pgen.1009192.s012.docx]

**S1 Table.** Genotypes of *Drosophila melanogaster* used in this study.

| **Figure panels** | **Experiments, Genotype** |
| --- | --- |
| ***Fig 1, S1 Fig, Fig2, S4 Fig*** | ***Triglyceride, trehalose, glucose and glycogen measurements***  ***Starvation and desiccation stress*** |
| 1A, S1A-S1C, 2A-2D, S4 | *w*; (*UAS-GFP* or *UAS-Toll^10b^*) / +; *r4-GAL4* / + |
| 1B | *w*; +/+; (*UAS-EGFP* or *UAS-Dif*) / *r4-GAL4* |
| 1C | *w*; +/+; (*UAS-EGFP* or *UAS-Toll^10b^*, *UAS-Dif^RNAi^*) / *r4-GAL4* |
| 1D-1F | *w*; +/+; *DrsGFP* |
| ***S2 Fig*** | ***RT-qPCR*** |
| S2B | *w*; +/+; *DrsGFP* |
| ***S3 Fig*** | ***cg-GAL4 driven Toll^10b^ phenotypes*** |
| S3A-S3G | *w*; *cg-GAL4* / (*UAS-GFP* or *UAS-Toll^10b^*) |
| ***Fig 3*** | ***RT-qPCR and triglyceride measurements, Lipin enzyme activity*** |
| 3B, 3C, 3E, 3G (left) | *w*; (*UAS-GFP* or *UAS-Toll^10b^*) / +; *r4-GAL4* / + |
| 3D, 3F | *w*; +/+; *DrsGFP* |
| 3G (right) | *w*; (*UAS-RFP* / *UAS-Toll^10b^*) / +; *r4-GAL4* / (*UAS-EGFP* or *UAS-LipinWT*) |
| 3H | *w*; (*UAS-RFP* / *UAS-Toll^10b^*) / +; *r4-GAL4* / (*UAS-EGFP* or *UAS-LipinWT*) |
| 3I | *w*; (*UAS-RFP* / *UAS-Toll^10b^*) / +; *r4-GAL4* / (*UAS-EGFP* or *UAS-mdy^HA^*) |
| 3J | *w*; (*UAS-RFP* / *UAS-Toll^10b^*) / +; *r4-GAL4* / (*UAS-EGFP* or *UAS-LipinWT*, *UAS-mdy^HA^*) |
| ***S5 Fig*** | ***RT-qPCR and triglyceride measurements*** |
| S5A | *w*; (*UAS-GFP* or *UAS-Lipin^RNAi^*) / +; *r4-GAL4* / + |
| S5B | *w*; (*UAS-GFP* or *UAS-mdy^RNAi^*) / +; *r4-GAL4* / + |
| S5C | *w*; (*UAS-GFP* or *mdy^QX25^*) / +; *r4-GAL4* / + |
| S5D | *w*; +/+; (*UAS-EGFP* or *UAS-LipinWT*) / *r4-GAL4* |
| S5E | *w*; (*UAS-GFP* or *P{EPgy2}mdy, CG13280*) / +; *r4-GAL4* / + |
| S5F | *w*; +/+; (*UAS-EGFP* or *UAS-mdy^HA^*) / *r4-GAL4* |
| S5G | *w*; (*CyO, GFP* or *mdy^QX25^*) / +; (*UAS-EGFP* or *UAS-mdy^HA^*) / *r4-GAL4* |
| S5H | *w*; (*UAS-RFP* / *UAS-Toll^10b^*) / +; *r4-GAL4* / (*UAS-EGFP* or *UAS-LipinWT*, *UAS-mdy^HA^*) |
| ***Fig 4 and S6 Fig*** | ***RT-qPCR and Western blot;***  ***Mass spectrometry and thin layer chromatography*** |
| 4B-4E, 4H, 4I,  S6A, S6B, S6D, S6E | *w*; (*UAS-GFP* or *UAS-Toll^10b^*) / +; *r4-GAL4* / + |
| 4F, 4G | *w*; +/+; *DrsGFP* |
| S3C | w; *UAS-RFP* / +; *r4-GAL4* / *UAS-EGFP* (RNA-Seq: Suzawa et al., 2019) |
| ***S7 Fig*** | ***RT-qPCR*** |
| S7A-S7C (left) | *w*; +/+; (*UAS-EGFP* or *UAS-Dif*) / *r4-GAL4* |
| S7C (right) | *w*; +/+; (*UAS-EGFP* or *UAS-Toll^10b^*, *UAS-Dif^RNAi^*) / *r4-GAL4* |
| ***Fig 5 and S8 Fig*** | ***RT-qPCR and Western blot*** |
| 5A, 5C, 5D, S8E | *w*; *Tub-GAL80ts* / (*UAS-RFP* or *UAS-Toll^10b^*); *r4-GAL4* / (*UAS-EGFP* or *UAS-Xbp1^RNAi^*) |
| 5B | *w*; +/+; *DrsGFP* |
| 5B (right) | *w*; +/+; (*UAS-EGFP* or *UAS-Toll^10b^*, *UAS-Dif^RNAi^*) / *r4-GAL4* |
| S8A-S8D | *w*; (*UAS-RFP* or *UAS-Toll^10b^*) / +; *r4-GAL4* / (*UAS-EGFP* or *UAS-SREBP1^RNAi^*) |
| ***S9 Fig*** | ***RT-qPCR*** |
| *S9A* | *y,w, UAS-Toll^10b^/* +; (*UAS-RFP* or *UAS-PEK^RNAi^*) / +; *r4-GAL4* / *+* |
| *S9B* | *y,w, UAS-Toll^10b^/* +; (*UAS-RFP* or *UAS-Atf6^RNAi^*) / *r4-GAL4* |
| ***Fig 6*** | ***RT-qPCR and electron microscopy*** |
| 6A-6D | *w*; (*UAS-GFP* or *UAS-Toll^10b^*) / +; *r4-GAL4* / + |
| ***Fig 7 and S10 Fig*** | ***RT-qPCR*** |
| 7A, S10A | *w*; (*UAS-RFP* or *UAS-Toll^10b^*) / +; *r4-GAL4* / *UAS-EGFP* (RNA-Seq: Suzawa et al., 2019) |
| 7B-7F, S10B | *w*; +/+ ; *r4-GAL4*, *Drs^∆7-17^* / (*UAS-EGFP* or *UAS-Toll^10b^*, *Drs^∆7-17^*) |
| 7B-7F, S10B | *w*; *Bom^∆55C^* / *Bom^∆55C^*; *r4-GAL4* / (*UAS-EGFP* or *UAS-Toll^10b^*) |
| 7B-7F, S10B | *w*; *Bom^∆55C^* / *Bom^∆55C^*; *r4-GAL4*, *Drs^∆7-17^* / (*UAS-EGFP* or *UAS-Toll^10b^*, *Drs^∆7-17^*) |
| 7C-7F | *w*; (*UAS-GFP* or *UAS-Toll^10b^*) / +; *r4-GAL4* / + |
| ***S11 Fig*** | ***Western blot and RT-qPCR*** |
| S11A | *w;* (*UAS-GFP* or *UAS-Toll^10b^*) / +; *r4-GAL4* / + |
| S11A | *w*; (*UAS-RFP* or *UAS-Pcyt1^RNAi^*) / +; *r4-GAL4* / (*UAS-EGFP* or *UAS-eas^RNAi^*) |
| S11A | *w*; (*UAS-RFP* or *UAS-Pcyt1RNA, UAS-Toll^10b^*) / +; *r4-GAL4* / (*UAS-EGFP* or *UAS-eas^RNAi^*) |
| S11B, S11C | *w*; (*UAS-RFP* or *UAS-Pcyt1^RNAi^*) / +; *r4-GAL4* / (*UAS-EGFP* or *UAS-eas^RNAi^*) |
| S11B, S11C | *w*; (*UAS-RFP* or *UAS-Pcyt1RNA, UAS-Toll^10b^*) / +; *r4-GAL4* / (*UAS-EGFP* or *UAS-eas^RNAi^*) |
| ***Fig 8*** | ***Western blot and RT-qPCR*** |
| 8A, 8B | *DiptLacZ*, *DrsGFP*, *y*,*w* / (*Y* or +); (*UAS-RFP* or *UAS-Toll^10b^*) / +; *r4-GAL4* / + |
| 8A, 8B | *DiptLacZ*, *DrsGFP*, *y*,*w* / (*Y* or +); *UAS-Pcyt1^RNAi^,* *UAS-Toll^10b^* / +; *r4-GAL4* / *UAS-eas^RNAi^* |
| 8C-8F | *w*; *UAS-RFP* / +; *r4-GAL4* / *UAS-EGFP* |
| 8C-8F | *w*; *UAS-Pcyt1^RNAi^* / +; *r4-GAL4* / *UAS-eas^RNAi^* |
